# Monti Sabatini and Colli Albani: the dormant twin volcanoes at the gates of Rome

**DOI:** 10.1038/s41598-020-65394-2

**Published:** 2020-05-26

**Authors:** F. Marra, C. Castellano, L. Cucci, F. Florindo, M. Gaeta, B. R. Jicha, D. M. Palladino, G. Sottili, A. Tertulliani, C. Tolomei

**Affiliations:** 1 Istituto Nazionale di Geofisica e Vulcanologia, Via di Vigna Murata 605, 00143 Rome, Italy; 2 Dipartimento di Scienze della Terra, Sapienza-Università di Roma, Piazzale Aldo Moro 5, 00185 Roma, Italy; 3 Department of Geoscience, University of Wisconsin-Madison, Madison, USA

**Keywords:** Natural hazards, Solid Earth sciences

## Abstract

This multi-disciplinary work provides an updated assessment of possible future eruptive scenarios for the city of Rome. Seven new ^40^Ar/^39^Ar ages from selected products of the Monti Sabatini and Vulsini volcanic districts, along with a compilation of all the literature ages on the Colli Albani and Vico products, are used to reconstruct and compare the eruptive histories of the Monti Sabatini and Colli Albani over the last 900 ka, in order to define their present state of activity. Petrographic analyses of the dated units characterize the crystal cargo, and Advanced-InSAR analysis highlights active deformation in the MS. We also review the historical and instrumental seismicity affecting this region. Based on the chronology of the most recent phases and the time elapsed between the last eruptions, we conclude that the waning/extinguishment of eruptive activity shifted progressively from NW to SE, from northern Latium toward the Neapolitan area, crossing the city of Rome. Although Monti Sabatini is unaffected by the unrest indicators presently occurring at the Colli Albani, it should be regarded as a dormant volcanic district, as the time of 70 kyr elapsed since the last eruption is of the same order of the longest dormancies occurred in the past.

## Introduction

**Figure 1:**
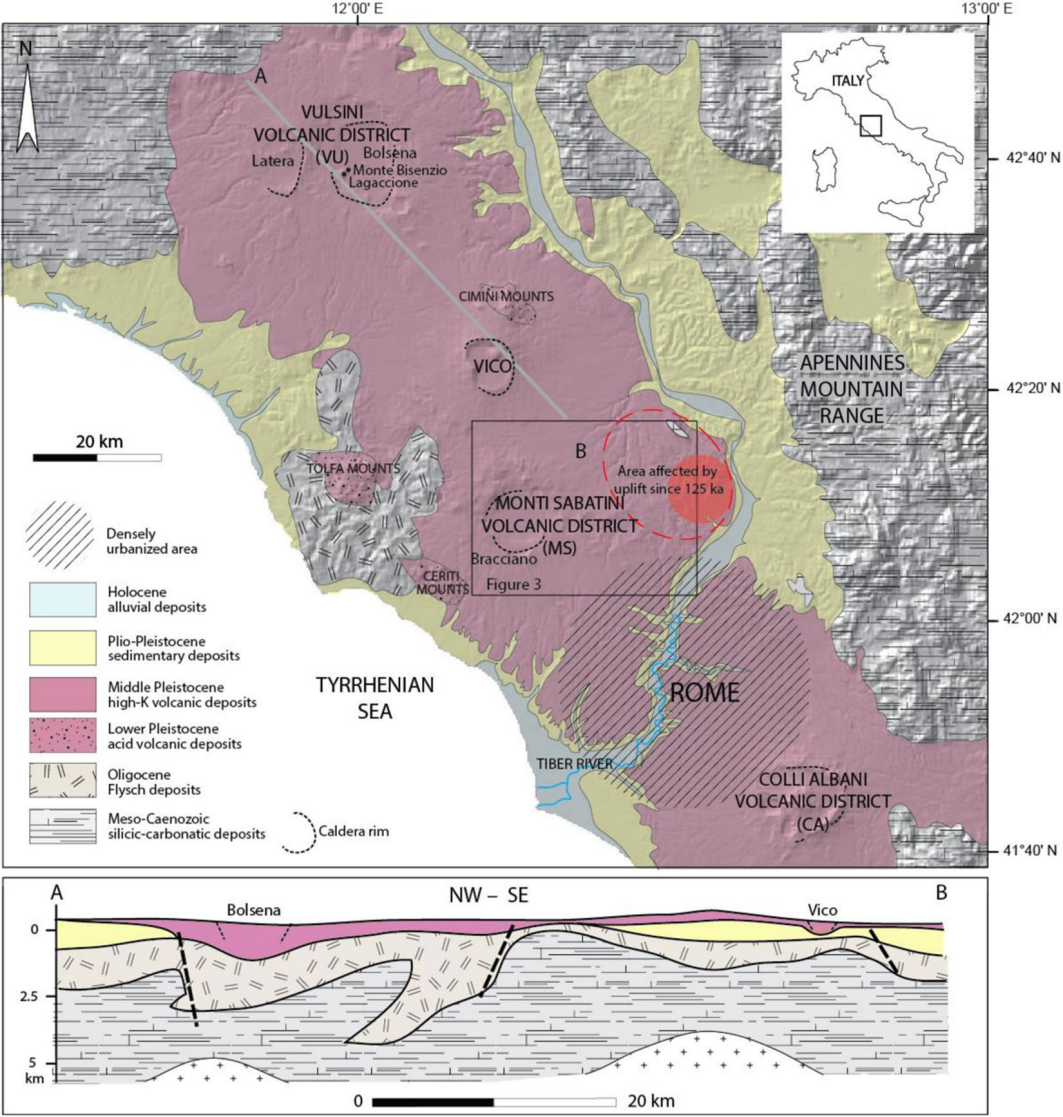
Geologic map drawn by F.M. showing the Roman volcanic province of northern-central Latium and the area affected by local uplift since 125 ka^1^. The deep geologic structure from gravimetry and borehole data by^7^ is re-drawn by F.M. for illustrative purpose in cross-section A-B. Background Digital Elevation Map (DEM) in this and in the following figures: TINITALY/01 square WA 6570, used with permission by the Istituto Nazionale di Geofisica e Vulcanologia, Rome.

The city of Rome was founded upon tuffaceous hills that are part of a thick pyroclastic plateau formed by the eruptions of two large volcanic districts: Monti Sabatini (MS) to the NW and Colli Albani (CA) to the SE (Fig. 1). A number of recent studies has ascertained the state of quiescence of the CA, given an average dormancy of 38.5 ± 1.5 kyr during the last 600 ka, and the occurrence of the most recent eruption at 36 ± 1 ka^1^ (and references therein). Moreover, a 20 year-long InSAR data record revealed ongoing inflation with maximum uplift rates >2 mm/yr in the area hosting the most recent (<200 ka) eruptive vents, suggesting that magma recharge is possibly occurring at depth^2^ (and references therein).

In contrast, no similar studies have been undertaken in the MS so far, precluding a reliable assessment of its actual state. However, a recent geomorphologic study on the fluvial terraces of the Tiber Valley found evidence of ca. 50 m of differential uplift occurred along the MS eastern sector in the last 125 kyr, concurrent with the most recent phase of eruptive activity from multiple vents^1^ (Fig. 1).

For this reason, a dedicated research project has been conducted to refine the MS eruptive history through a set of new ^40^Ar/^39^Ar age determinations, and to assess possible active soil deformation, including seismotectonic processes and vertical movements, through InSAR analysis.

The analysis of the regional seismicity, comparing instrumental and historical records for the four volcanic districts of Latium, is provided aiming at highlighting possible unrest indicators, as recently shown for the CA^2^.

## Geologic setting

**Figure 2:**
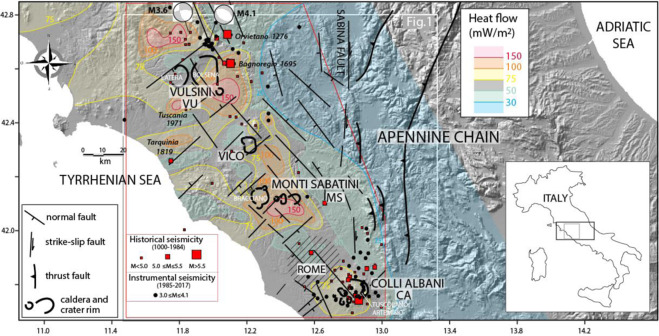
Structural sketch for central Italy drawn by F.M., showing the volcanic districts of the Roman Province and the main tectonic structures (original elaboration based on data from^6^); the regional heat flow distribution is also shown (original elaboration based on data available at Istituto di Geoscienze e Georidsorse - C.N.R.; http://217.174.128.43/wm_geothopica/map.phtml?winsize=large&language=it&config = ), as well as the historical and instrumental seismicity in the northern-central Latium region. Red squares mark the occurrences of historical earthquakes between 1000 and 1984 AD from the CPTI Catalogue^14^. Black dots indicate the instrumental seismicity between 1985 and 2018 (http://cnt.rm.ingv.it). The TDMT of two earthquakes (M4.1 and M3.6) occurred in 2016 in the northern area are available at http://iside.rm.ingv.it/tdmt. Background Digital Elevation Map (DEM): TINITALY/01 square WA 6570, used with permission by the Istituto Nazionale di Geofisica e Vulcanologia, Rome.

The MS belongs to the highly potassic Roman Magmatic Province^3^, which originated in the area comprised between the Apennine orogen and the Tyrrhenian Sea coast during Pleistocene times. This back-arc geodynamical domain^4,5^ was affected by extensional tectonics as a consequence of the NE retreat of the slab since Messinian times. Principal NW-SE trending normal faults and NE-SW transfer faults^6^, bordering the sedimentary basins, favored the uprising of magma from the upper mantle. Consequently, acid volcanism during Pliocene-Lower Pleistocene^7,8^, followed by the high-K volcanism of the Roman Province occurred in the Middle Pleistocene^3,8^. Concurrent strike-slip faulting associated with a N-S shear zone (Sabina Fault Zone^9^) (Fig. 2) also superposed the extensional regime in this region^10–13^.

An interpretation of the subsurface geology of this region, based on gravimetric and borehole data^7^, is shown in the cross-section of Fig. 1. A variably thick succession of flysch deposits overlies and is occasionally interlayered with the uppermost portion of a Meso-Cenozoic carbonate bedrock, as a consequence of thrust faulting linked with the compressive stages of the tectonic evolution of the Apennine chain. Successive extensional tectonics caused the dismembering of this substrate in horst and graben systems and the deposition of significant thickness of Plio-Pleistocene marine deposits within the subsiding sedimentary basins. The occurrence of Plio-Pleistocene intrusive bodies has been inferred at ca. 3500 m of depth below the main acid volcanic complexes of Tolfa, Ceriti and Cimini. Also, a very variable, both in thickness and lithology, cover of Middle-Upper Pleistocene potassic volcanics of the Roman Province, particularly thickened within the collapsed calderas (e.g., Bolsena, Latera, Vico, Bracciano, Tuscolano-Artemisio), fills the sedimentary basins and extends on the structural heights.

The map of the heat flow by the Istituto di Geoscienze e Georisorse of C.N.R. - Italy, shows two maxima of 150 mW/m^2^ in correspondence of the Vulsini Volcanic District (VU) and the MS, and a third relative maximum of 100 mW/m^2^ in correspondence of the Vico volcanic district (Fig. 2). A drastic drop SE of the MS characterizes the area of Rome, whereas a moderate increase of the heat flow occurs below the CA, with a relative maximum of 75 mW/m^2^.

## Seismic features

From a seismic point of view, northern Latium is a low- to moderate seismicity region, even if it can suffer strong shaking from external high-seismicity areas, like those located along the Central Apennines. The Italian Parametric Catalogue of Earthquakes (CPTI15^14^) reports that the maximum event occurred in northern Latium is the so-called 1695 Bagnoregio earthquake (I_0_ 8–9, M_w_ 5.8), whose epicenter was located at the eastern margin of the VU, along the western side of the Tiber valley. However, the effective size of this event has been recently questioned^15^.

The historical record of the seismicity of the Tyrrhenian side of the studied region is fragmentary. There, two particular cases emerge: i) the doubtful 1819 Tarquinia earthquake (M 5.1), which is poorly documented; ii) the 1971 Tuscania earthquake (I 8–9, M_l_ 4.8), unique and very damaging event in the area^16^, which caused destruction and resulted in thirty victims in the town of Tuscania. The only contemporary seismological study of this earthquake^17^ attributes the unusually significant damage to its shallow focal depth (4.1 km), along with the poor lithological characteristics and the inadequate building stock of the historical center of Tuscania. No evidence has been provided so far to identify the seismogenic source of this earthquake; however, the low magnitude and the shallow depth, along with the rapid intensity attenuation with distance, point to a small-size, local structure located within the sedimentary bedrock. A recent reappraisal^18^, based on a detailed study of the damage, suggests an I_max_ 8 in the European Macroseismic Scale and confirms the remarkable vulnerability of local buildings at the time of the earthquake.

Most of the micro-seismicity of this sector of central Italy is concentrated in the VU: the instrumental seismicity shows that low-magnitude and shallow earthquakes are usually confined between 3 and 8 km of depth. The shallowest earthquakes are probably related to geothermal activity (hydrofracturing associated with well reinjection tests^19^). In the VU area, two focal mechanisms are available^20^; in particular, the revised mechanism of a M4.1 earthquake occurred in 2016 shows normal faulting along NW-trending fault planes. In the central VU, and along the Tyrrhenian Sea coast, seismicity becomes even more sparse and infrequent. It is worth noting that south of Rome, the features of seismicity change dramatically in frequency and magnitude when compared with the northern Latium region (Fig. 2). Actually, frequent damaging earthquakes occurred in historical times in the CA (CPTI15^14^), and several low-to-moderate magnitude seismic swarms in the instrumental era (http://cnt.rm.ingv.it). The most probable reason for such different regime for the relatively low-seismicity associated with the VU and MS, which cannot be related to significant gaps in the instrumental network, can be found in the high local heat flow (Fig. 2). High heat flow likely hampers strong frictional slip at depth^15^. However, the lack of seismicity in the upper crust below the MS, unlike at CA^2^, suggests the absence of shallow magma reservoirs.

### The MS eruptive history

**Figure 3:**
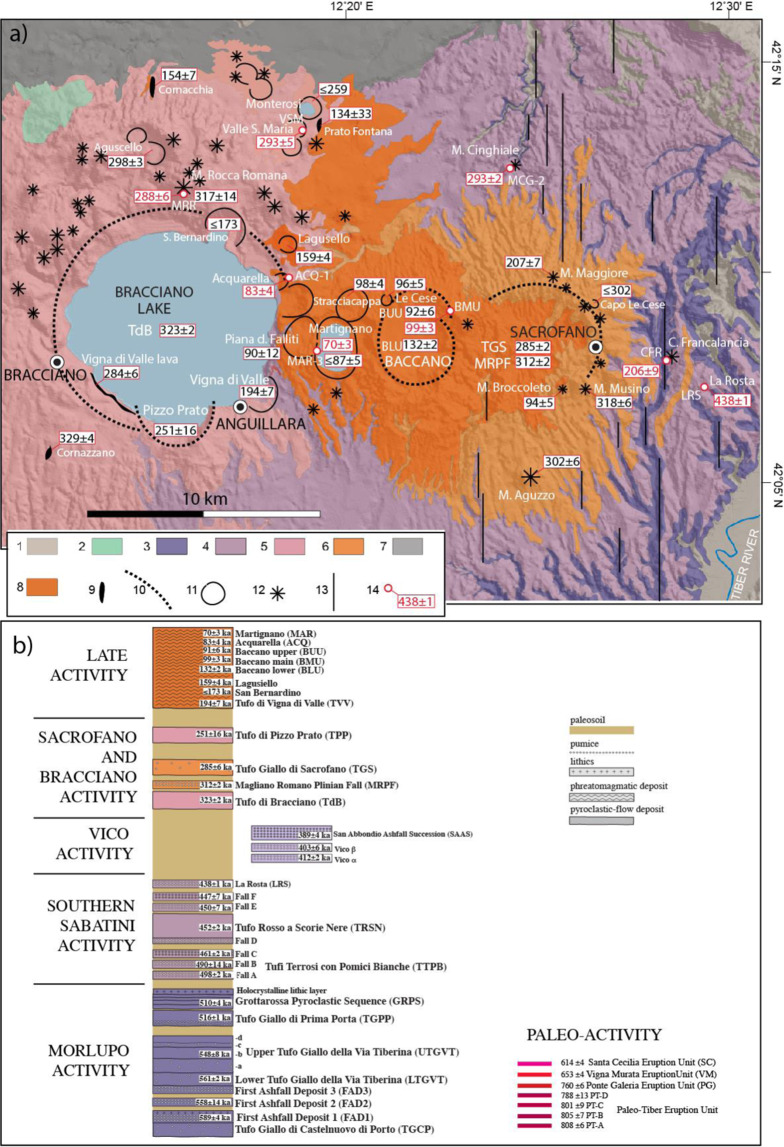
(**a**) Geological map of the MS drawn by F.M., showing the locations of the recently dated samples^1^ (and this work) and a summary of previous age determinations on the <350 ka eruptive products (see text for detail and references). Legend: **1** - Sedimentary terrains; **2** - Monti Ceriti-Tolfetano-Manziate lava domes (Pliocene); **3** – products of the Morlupo source area (0.6-0.5 Ma); **4** – products of the Southern Sabatini source area (0.5 − 0.4 Ma); **5** Bracciano Caldera products (0.3-0.2 Ma); **6** - Sacrofano Caldera products (0.3-0.2 Ma); **7** – Tufo Rosso a Scorie Nere Vicano Unit (0.15 Ma); **8** - Late Sabatini activity (<0.15 Ma); **9** - lava flow; **10** - Caldera rim; **11** - Crater rim; **12**- Scoria cone; 13 - morpho-structural lineament; 14 - dated sample (age in ka). (**b**) Sketch composite stratigraphic log of MS. Background Digital Elevation Map (DEM): TINITALY/01 square WA 6570, used with permission by the Istituto Nazionale di Geofisica e Vulcanologia, Rome.

The MS activity has been grouped into six main eruptive phases (see the stratigraphic scheme in Fig. 3b and detailed description in Suppl. Mat. #1). These were characterized by volumetrically dominant explosive eruptions, ranging from hydromagmatic to Plinian and large pyroclastic flow-forming events (Volcanic Explosivity Index or VEI up to 4–5), with erupted magma volumes up to tens of km^3^ (Dense Rock Equivalent) for individual events, and subordinate effusive episodes^1,21–24^.

Numerous geochronologic constraints to these phases of activity were provided in previous literature through ^40^Ar/^39^Ar ages of the volcanic products^1,23–25^, which are reported here calibrated (or recalculated) according to the standard ages of 28.201 Ma for the Fish Canyon Tuff sanidine^26^, and 1.186 Ma for the Alder Creek sanidine^27^.

### Sample selection criteria

Stratigraphic and geomorphologic investigations in the study area focused on the undated volcanic centers and eruptive products at the MS and VU to identify the most recent eruptive events. Ten samples were selected for ^40^Ar/^39^Ar dating. Results for three of these samples have been already published^1^, while the remaining seven are unpublished. We combine new data with a review of previous literature data, allowing for a comprehensive overview of the phases of eruptive activity in the last 350 kyr.

In particular, for MS, we selected the products of four phreatomagmatic centers (i.e., Baccano, Acquarella, Martignano, Valle Santa Maria, Fig. 3) that, based on stratigraphic and geomorphologic evidence, are inferred as the youngest among those previously undated. Due to the widespread occurrence of accessory lithics in these products and the difficulty to distinguish the syn-eruptive juvenile fraction (e.g.^28^), in some instances we have chosen to date single crystals collected from the ash matrix of pyroclastic-surge deposits. The youngest crystal population in these samples, if not occurring unambiguously in the juvenile fraction, at least provides a *post-quem* (maximum) age for the eruptive event. With this aim, we investigate the compositional and depositional features of the dated phreatomagmatic products, both at the outcrop scale and at the optical microscope, in order to discriminate the juvenile vs. antecrystic vs. xenocrystic origin of the dated crystals.

In addition, we have dated three lava flows (i.e., Monte Rocca Romana-MRR, Casale Francalancia-CFR, Monte Cinghiale-MCG) representing a transitional stage between the highly explosive phases of the Bracciano and Sacrofano calderas and the most recent phreatomagmatic phase occurred at Baccano and nearby centers^23^. Two of these lava flows (MRR, MCG) show highly vesicular texture, unsuitable for step-heating analysis on the groundmass. Therefore, leucite phenocrysts have been extracted from these samples for single-crystal total fusion analysis.

Finally, we have dated a Plinian fall deposit collected at La Rosta locality (LRS), aiming at better constraining the already recognized, long period of dormancy at the MS from 447 ± 5 to 329 ± 5 ka^24^.

The integrated geochronologic dataset presented in this paper can be considered as representative of all the volumetrically significant eruptions occurred at the MS since the onset of the early explosive activity at 589 ± 4 ka, including the previously identified centers of late phreatomagmatic activity^23,29–31^.

In contrast, a less detailed geochronological dataset is available so far for the VU eruptive activity, broadly spanning the 600–100 ka time interval (see^32^ and reference therein). As part of an ongoing research project addressing the whole Roman Province, here we present two new age determinations aimed at defining the most recent VU eruptive events, up to now represented by a lava flow from the Bisentina Island in Lake Bolsena caldera (K/Ar age of 127 ± 2 ka^33^). In this regard, we sampled the eruptive products of the Monte Bisenzio spatter cone and the Lagaccione maar-tuff ring, both located SW of Bolsena lakeshore and supposedly being among the most recent eruptive centers, based on stratigraphic evidence and well preserved morphologies^34^. In both cases, the lack of K-bearing phenocrysts suitable for dating in the associated spatter clasts led us to date a lava dyke cutting the Monte Bisenzio cone (sample BSZ-LD) and the scoria lapilli from the phreatomagmatic surge deposits directly overlying spatter fall deposits at Lagaccione (sample LGC-2).

## Results

### A-InSAR analysis

**Figure 4:**
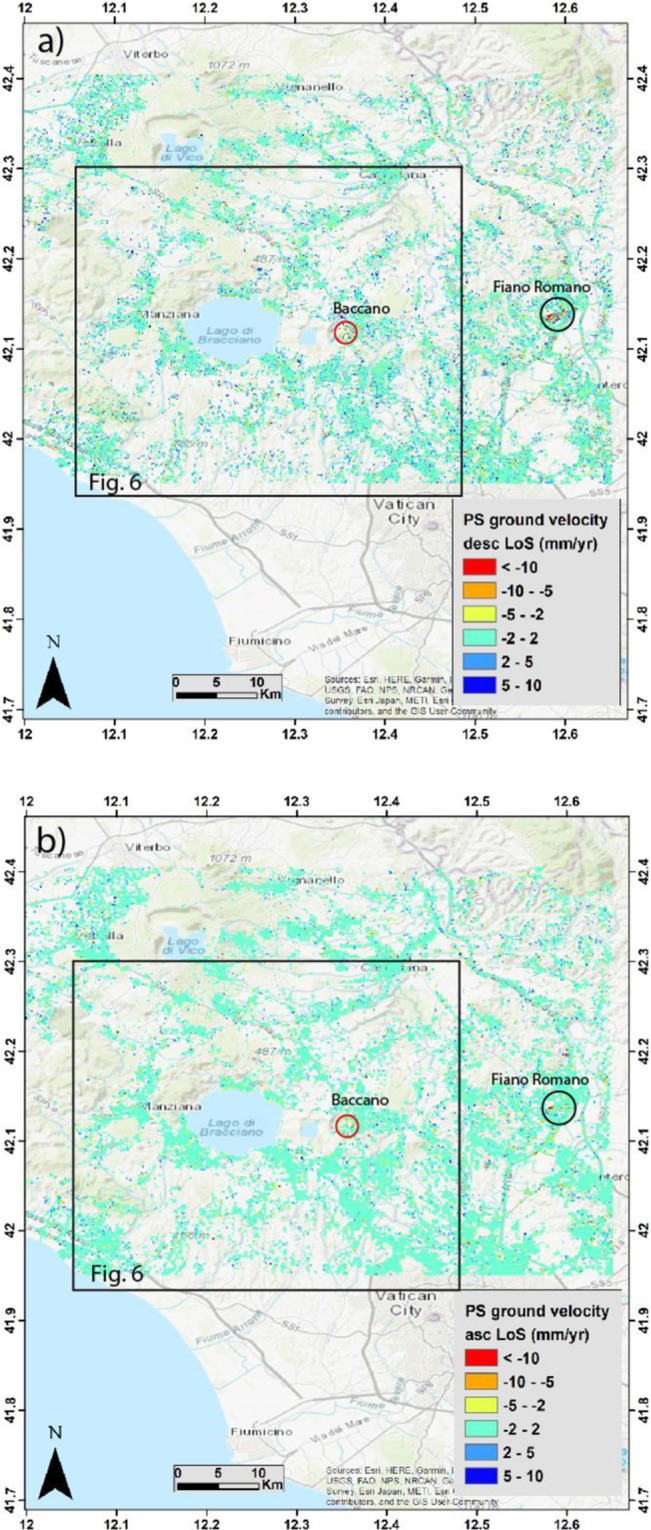
Descending (**a**) and ascending (**b**) mean ground velocity map of the MS area and surroundings from PS technique. Background Digital Elevation Map (DEM): TINITALY/01 square WA 6570, used with permission by the Istituto Nazionale di Geofisica e Vulcanologia, Rome.

The Interferometric synthetic-aperture radar (In-SAR) analysis is a well-accepted monitoring method to investigate ground surface movement. In order to detect coherent vertical movements, the results obtained using the Permanent Scatterers (PS) approach (see methods) along the ascending and descending orbits must be compared (Fig. 4).

**Figure 5:**
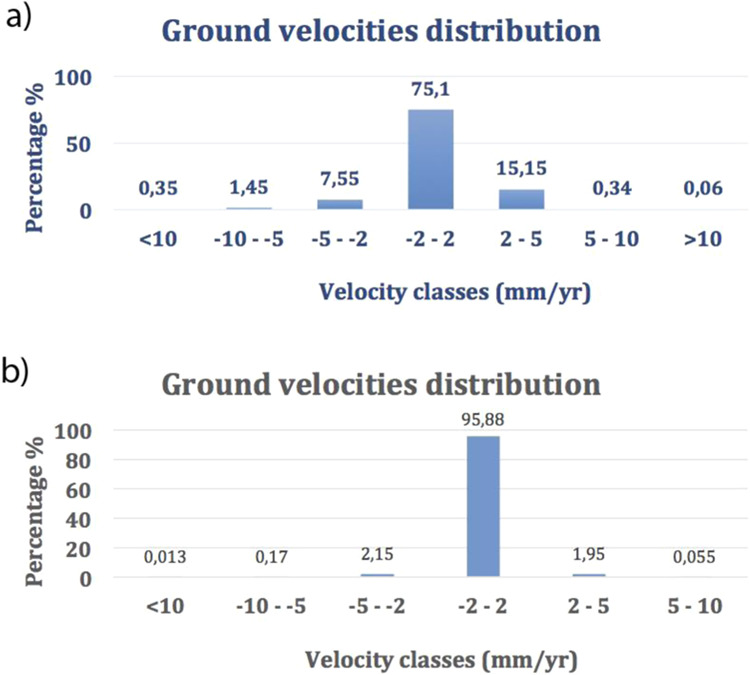
Ground velocity distribution: (**a**) S1 Descending orbit result; (**b**) S1 Ascending orbit result.

As an overall feature of the MS area and surroundings, the color pertaining to vertical movements comprised between +2 and −2 mm/yr (i.e., within or slightly above the analytical error) is largely prevailing in the retrieved ground velocity maps. The proportion of the areas affected by each range of velocity values in the maps is reported in Table 1 and Fig. 5 for both the descending and ascending orbits.

**Table 1:** Statistics of ground velocity distribution (a) S1 Descending orbit result; (b) S1 Ascending orbit result.

Classes Range	Percentage %	Mean value	Standard Deviation	Nr. of pixels
(a)				
<10	0.35	−13.5	3.33	64
−10–5	1.45	−6.61	1.3	310
−5–2	7.55	−2.95	0.78	1908
−2–2	75.1	0.21	1	18955
2–5	15.15	2.91	0.73	3817
5–10	0.34	6.07	0.99	293
>10	0.06	12.3	1.43	9
(b)				
<10	0.013	−11.3	1.12	5
−10–5	0.17	−6.5	1.33	41
−5-2	2.15	−2.78	0.67	507
−2–2	95.88	0.11	0.68	22527
2–5	1.95	2.58	0.58	457
5–10	0.055	6.42	0.65	13

However, rapid and very concentrated subsidence phenomena, showing negative values of the velocities higher than −10 mm/yr, are observed in correspondence of the industrial area near Fiano Romano and in the reclaimed marshy plain within the Baccano caldera (black and red circles in Fig. 4, respectively). This marked subsidence is readily associated with water exploitation for anthropic use (i.e., industrial and agricultural use, respectively). In contrast, positive and negative velocities >2 mm/yr are intermingled throughout the investigated area, without defining any coherent framework. The lack of any significant displacement pattern suggests that the investigated area should be considered generally stable from a volcano-tectonic point of view.

**Figure 6:**
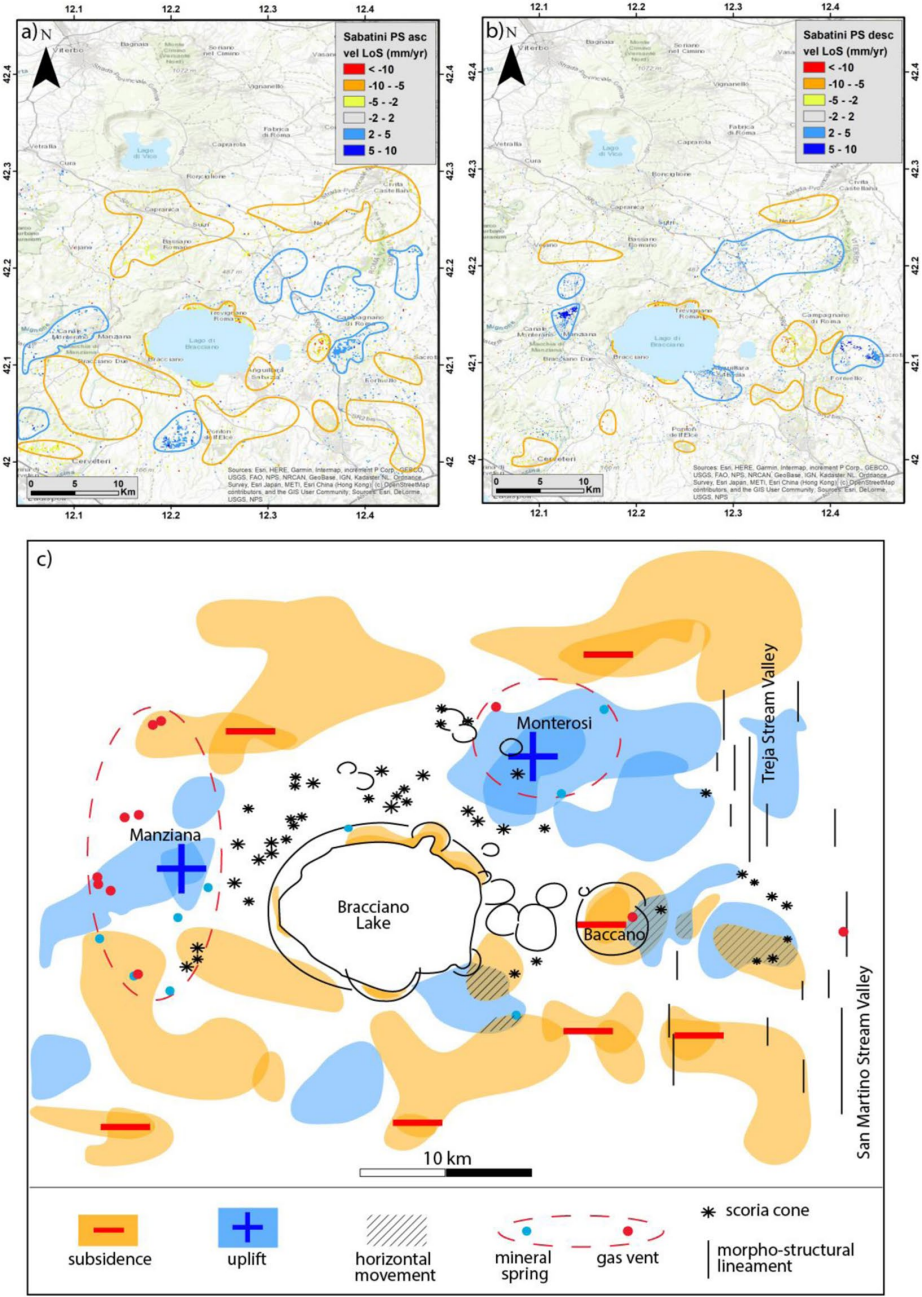
Results of the SAR stack processing in a smaller sector hosting the MS. Sectors characterized by marked prevalence of negative (encircled by yellow lines) and positive (encircled by blue lines) velocities are identified in the ascending (**a**) and descending (**b**) orbit maps. Background Digital Elevation Map (DEM): TINITALY/01 square WA 6570, used with permission by the Istituto Nazionale di Geofisica e Vulcanologia, Rome. (**c**) Sectors characterized by coherent vertical movements are reported with yellow (negative velocities) and blue (positive velocities) shading; sectors characterized by opposed velocities, which are therefore affected by prevailing horizontal displacement, are reported with oblique dashing.

In order to investigate in more details the volcanic area, we have repeated the SAR stack image processing in a smaller sector hosting the MS (Fig. 6).

This new A-InSAR processing outcomes highlighted a slightly more coherent picture with respect to the distribution of the negative and positive displacement velocities exceeding 2 mm/yr. This may be the result of different reference points chosen by the software for each sub-patch, in which it divides the full area of interest, passing from the larger to the smaller area. In Fig. 6a,b, PS characterized by velocities <+2/−2 mm/yr are hindered in order to highlight the distribution of the higher displacement velocities. Results for the two orbits are compared for consistency and the output is reported in Fig. 6c.

Although the results obtained in the smaller area should be regarded with caution, as they may merely be indicative of possible trends, we note a meaningful pattern, consistent with the geologic-structural features of this sector. In particular, the general subsidence in the wider area around the Bracciano Lake and the higher negative velocities along the lakeshore finds straightforward correlation with the large decrease of the lake level occurred over the past several years. After a period of reduced winter rainfall, the water lake reached −198 cm with respect to the hydrometric zero in the summer of 2017. Due to this hydrologic crisis, the local authority ordered the suspension of the withdrawal from Lake Bracciano, one of the Rome’s water reserve, adducted by the Peschiera aqueduct. The general lowering of the water table in this area is therefore the most likely cause for the observed subsidence. We remark that the highest negative velocities >10 mm/yr are concentrated within the Baccano caldera also in the results for the SAR stacking in the smaller area, consistent with the results obtained for the larger one.

Moreover, two uplifting sectors located north of the Bracciano caldera are also highlighted in Fig. 6c. Notably, they are associated with areas where hydrothermal manifestations concentrate (encircled by the dashed red lines), at Manziana and Monterosi. Uplift in these small sectors is therefore explainable by uprising of fluids associated with late hydrothermal activity.

### ^40^Ar/^39^Ar ages

The results of the ^40^Ar/^39^Ar analyses are summarized in Table 2. Ages in Table 2 and throughout the text are reported at 2σ analytical uncertainty and do not include uncertainty in the flux monitor age and/or decay constants. Full analytical data including methods are provided in Supplementary Material #2; the detailed description of the dated samples is provided in Supplementary Material #3. ^40^Ar/^39^Ar data are discussed below.

**Table 2:** ^40^Ar/^39^Ar ages and coordinates of the samples dated for the present study and (*) in^1^. **stratigraphically inconsistent age, N: number of crystals included in the weighted mean, SH: step heating experiment.

Sample	Unit/Eruptive center	Long.	Lat.	Age ± 2σ (ka)	N	phase
	MS					
MAR-3*	Martignano upper unit (phreatomagmatic surge)	12°18′24″E	42°06′11″N	70.0 ± 3.3	14/14	San
ACQ*	Acquarella (phreatomagmatic surge)	12°17′32″E	42°08′27″N	82.5 ± 4.4	5/17	San
BMU*	Baccano final main unit (phreatomagmatic surge)	12°22′36″E	42°07′34″N	99.3 ± 2.7	5/21	San
CFR-LF	Casale Francalancia lava flow	12°29′08″E	42°06′44″N	206.3 ± 8.8	SH	Lct
MRR	Monte Rocca Romana lava flow	12°14′06″E	42°10′09″N	288.1 ± 5.9	22/22	Lct
VSM	Valle Santa Maria (phreatomagmatic surge)	12°17′41″E	42°11′40″N	292.7 ± 4.7	4/17	San
MCG	Monte Cinghiale lava dome	12°24′18″E	42°11′01″N	292.7 ± 1.8	18/18	Lct
LRS	La Rosta Plinian fall	12°30′24″E	42°06′00″N	437.7 ± 1.2	21/21	San
	**VU**					
BSZ-LD	Monte Bisenzio lava dyke	11°52′25″E	42°34′26″N	111 ± 18	SH	Ground-mass
LGC-2	Lagaccione (phreatomagmatic surge)	11°51′44″E	42°33′54″N	*215.7* ± 4.2**	16/18	San

### Petrography

Results of thin section observations focused on the phreatomagmatic products dated in this work and in^1^ are summarized in Table 3. The implications for the interpretation of the ^40^Ar/^39^Ar ages are discussed below.

**Table 3:** Summary of petrographic observation in thin section of representative samples of the late activity at MS and VU. cpx: clinopyroxene; Ol: olivine; lct: leucite; san: sanidine. (1) Data from:^53,54^; (2) data from^31,34^. ND: not determined.

Sample	Macroscopic aspect	Texture of juvenile clasts	Phenocrysts	Groundmass and micro-phenocrysts	Glass composition^(1)^	Bulk composition^(1,2)^	Matrix lithics and xenocrysts
MAR-3	Single scoria with mingling domains enclosed in a thin matrix rim	Porphiritic, low degree of vesicularity, small vesicles	San, lct, cpx, dark mica, spinel	Glassy, with color variations	Bimodal:phonotephrite and phonolite	Phonolite	San-bearing ash matrix; ol xenocrysts
ACQ	Clast-supported, consolidated	Porphiritic, small vesicles	Cpx, dark mica	Glassy to criptocrystalline	Phonolite	Phonolite	Scarce, turned to zeolite, with abundant loose lct, san, cpx, dark mica, garnet, amph; abundant accessory, scarce accidental carbonate
BMU	Matrix- supported, consolidated	Porphiritic, poorly vesicular, small vesicles	San, dark mica	Glassy	Phonolite	Phonolite	Abundant, rich in calcite, with abundant loose lct, san, cpx, dark mica; abundant accidental carbonate, scarce accessory
VSM	Clast- to matrix- supported, consolidated, with abundant fine scoria lapilli	Porphiritic, poorly vesicular, criptocrystalline groundmass	Scarce lct and green cpx	Criptocrystalline with abundant lct frequently with star-like habit, scarce green cpx, rare glass	ND	Phonotephrite	Scarce, with abundant loose lct, san, cpx; frequent lct-bearing accessory
LGC	Matrix- supported, consolidated	Porphiritic, highly vesicular	Ol, cpx	Glassy with rare feldspars	ND	Trachybasalt	Abundant, rich in loose mafic minerals, frequent san, scarce lct; rare accessory

Detailed compositional and depositional features of the dated deposits are described in Supplementary Material #3 (Sample description), along with TAS composition of the lava flows.

## Discussion

### MS eruption ages

Here we discuss the significance of the new ^40^Ar/^39^Ar ages in the frame of the MS activity history. The age of 438 ± 1 ka for the Plinian fall deposit identified at La Rosta locality (in the eastern MS), puts further constraints to the dormancy interval that followed the major explosive activity of the Southern Sabatini^22,24^ and preceded the onset of the Bracciano and Sacrofano activities^23^ (see section 4.2). Moreover, this deposit possibly represents the proximal equivalent of a widespread tephrostratigraphic marker, which in the coastal area of Rome is an *ante-quem* terminus (minimum age) for glacial termination V, as evidenced by the local MIS 11 aggradational succession (San Paolo Formation^35^).

The age of 288 ± 6 ka for the Monte Rocca Romana edifice contributes to the definition of the peri-caldera activity phase occurred at a number of eruptive centers (mainly scoria cones) located along the northern rim of the Bracciano caldera (Fig. 3), following the 323 ± 2 ka Plinian eruption^36^. Similarly, the age of 292 ± 2 ka for the Monte Cinghiale lava dome implements the chronological record of the Sacrofano eruptive activity^23^, predating the major Tufo Giallo di Sacrofano caldera-forming event. To the east of the Sacrofano caldera, the age of 206 ± 9 ka for the Casale Francalancia lava flow, coeval with the Monte Maggiore scoria cone (207 ± 7 ka^23^), suggests a common tectonic control on the magma feeder system by fault activity along two major, *en-echelon* N-S lineaments, previously identified in the streambeds of the Treja and San Martino hydrographic networks^10^ (Fig. 3).

Concerning the widespread phreatomagmatic activity, the Valle Santa Maria pyroclastic surge, dated in the present work, as well as two other phreatomagmatic products (Acquarello surge and Baccano main unit) dated in^1^, revealed a wide range in dates, which is likely due to the presence of antecrysts and/or xenocrysts. In these cases, the eruption ages are assessed based on the youngest populations of crystals (either sanidine or leucite; Table 2) extracted from both scoria clasts and the enclosing matrix of bulk samples. Therefore, these ages should be regarded with caution, as they may represent *post-quem* terminus (maximum) ages. For an in-depth discussion of this key issue see^37^.

The new age for the Valle Santa Maria center (~292 ka), along with that previously obtained for the nearby Monterosi center (~257 ka^23^), would conflict with the reported occurrence of accessory lithics from the ~152 ka Vico C ignimbrite^23,38^. Indeed, observation in thin section of the dated sample VSM shows the occurrence of leucite in the juvenile fraction, which could not be separated in clean grains of datable size, whereas the youngest population yielding 292.7 ± 4.7 ka consists of loose sanidine crystals separated from the matrix (Table 2), which may represent xenocrysts. In contrast, in the Monterosi case, a supposedly juvenile fraction was selected for dating^23^. Actually, the lack of loose crystals younger than 292 and 257 ka, respectively, in the matrix of these products, does not support the entrapment of tuff lithics as young as 152 ka from the pre-eruptive substrate. Therefore, the previous attribution to Vico C of these tuff inclusions needs further checking. If we rely on the ages obtained for the two centers (located to the north of Bracciano Lake), they would point out the occurrence of a phreatomagmatic activity concomitant to the above-mentioned peri-caldera Strombolian and effusive activities (e.g., Monte Rocca Romana, 288 ± 5 ka) along the northern rim of the Bracciano caldera since ~320 ka.

The most recent activity in the northern MS is documented by a poorly constrained ^40^Ar/^39^Ar age of 134 ± 33 ka and a more precise age of 154 ± 7 ka, respectively for the Prato Fontana and Cornacchia lava flows^31^, cropping out a few km south of Valle Santa Maria. This activity shortly preceded the onset of the most recent eruptive period in the whole MS at 131 ± 2 ka in the Baccano-Martignano area^23^. In this regard, the age of 99.3 ± 2.7 ka for sample BMU, based on the 5 youngest crystals out of 21 (Table 1), falls in between the Baccano lower (131 ± 2 ka) and upper (91 ± 6 ka) units^23^, thus providing mutually consistent constraints to the Baccano eruptive activity.

The age of sample ACQ-1 is based on the 5 youngest crystals out of 17 from the matrix of the phreatomagmatic deposits. Their weighted mean age of 82.5 ± 4.4 ka represents the second youngest age, after Martignano, in the MS. On the other hand, the age of 70.0 ± 3.3 ka yielded by the Martignano upper surge deposit (MAR-3) bears on 14 out of 14 dated sanidine crystals, thus supporting their juvenile nature, despite their occurrence as loose crystals in the matrix of a phreatomagmatic deposit. This age value is confidently assumed as the reliable eruption age, thus accounting for the youngest event ever identified in the MS.

The results from the above three samples (BMU, ACQ, MAR-3) from Baccano and nearby phreatomagmatic centers, combined with those previously obtained for the late MS activity^23^, are summarized in Fig. 6. Consistent with morpho-stratigraphic evidence, we notice an eruptive cluster in the Baccano-Martignano area, spanning ~131-70 ka, which represents the most recent activity phase at MS. Specifically, the activity of the Martignano center, a composite maar-tuff ring, is characterized by three eruption units, separated by incipient paleo-soils, spanning the interval from 87 ± 5 ka (youngest crystal age for the lowest unit^23^) to 70 ± 3 ka^1^.

### VU eruption ages

Up to now, the most recent dated product at VU was the tiny lava flow exposed on the Bisentina Island, with a K/Ar age of 127.4 ± 1.8 ka^33^. Our attempt to date spatter clasts from the Monte Bisenzio eruptive center failed due to the lack of suitable K-bearing crystals and the highly vesicular groundmass. Thus, we sampled the associated lava dyke and dated the groundmass via a step-heating experiment, which yielded a plateau age of 111 ± 18 ka (MSWD = 0.85) with an isochron that has a ^40^Ar/^36^Ar intercept within uncertainty of the atmospheric value (see Supplemental data) (BSZ-LD; Table 2). This age possibly accounts for the youngest documented volcanic activity at VU (or, at least, considering the relatively large uncertainty, in the same range than previously known).

For the Lagaccione maar-tuff ring, we dated loose K-bearing feldspar crystals from the ash matrix of phreatomagmatic surge deposits on top of the co-eruptive spatter fall deposit. The weighted mean age of 215.7 ± 4.2 ka (based on 16 out of 18 dated crystals; LGC-2, Table 2) conflicts with the stratigraphic position of the Lagaccione products on the Onano eruption products, which in turn occur on top of the Grotte di Castro pyroclastic-flow deposit, recently dated at 185 ± 9 ka^39^. This stratigraphic inconsistency might be by-passed only if we consider the youngest crystal age obtained (199 ± 27 ka) at its youngest extreme (172 ka). Likely (as typical in bulk samples of phreatomagmatic products) this age measurement is strongly affected by extensive (or even total) xenocryst contamination, and thus is unreliable for the eruption age assessment.

### Implemented reconstruction of the MS eruptive history

**Figure 7:**
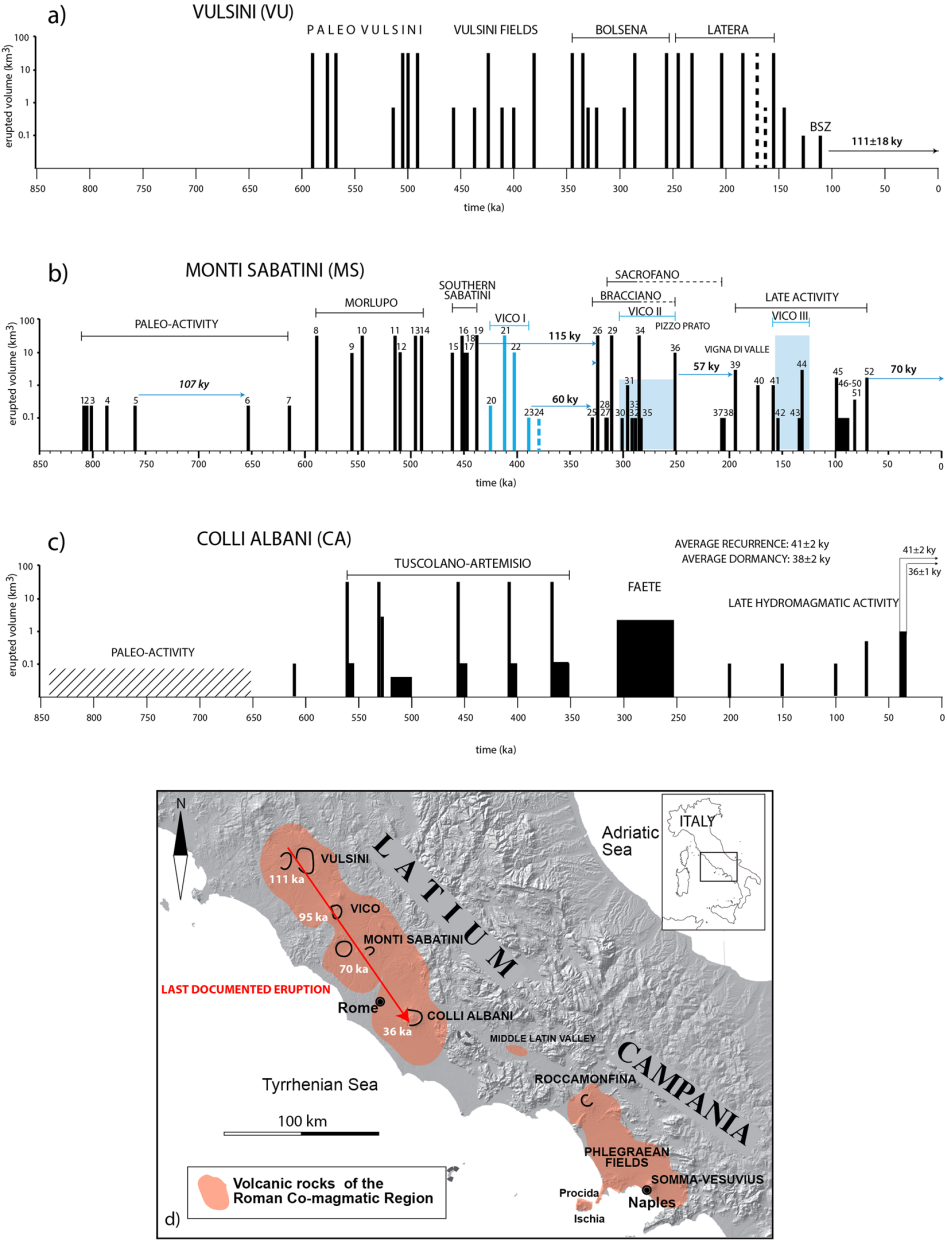
(**a**) VU eruptive history, based on a combined dataset of K/Ar and ^40^Ar/^39^Ar literature data^32^ (and references therein), new ^40^Ar/^39^Ar ages from^37^. Age of sample BSZ performed in this study is also reported; (**b**) MS eruptive history. Mean ages of all the available (fifty-two) ^40^Ar/^39^Ar ages from the literature and the present study are reported without the analytical errors as vertical bars (blue color indicates those from Vico). Height of the bars (in logarithmic scale) is proportional to a qualitative estimation of the erupted volumes, based on areal distribution and thickness of deposits and crater/edifice size. Horizontal arrows highlight the main dormancies (see text for discussion). Progressive numbering as in Table 4; (**c**) CA eruptive history (original dataset in^37^). Vertical bars report individual major eruptive events, while boxes represent long-lasting (grey fill) or poorly constrained (oblique fill) activity phases; (**d**) Overview of the Quaternary potassic Roman Co-magmatic Region suggesting a progressive NW to SE extinguishment of eruptive activity at the volcanic districts of Latium (based on the available age constraints). Background Digital Elevation Map (DEM): TINITALY/01 square WA 6570, used with permission by the Istituto Nazionale di Geofisica e Vulcanologia, Rome.

An updated summary of the MS eruptive history, based on all the available (25) ^40^Ar/^39^Ar ages from the literature and the present study is shown in Fig. 7b and detailed in Table 4. Although still incomplete, this dataset can be considered as broadly representative of the whole MS activity record, allowing an overview of the timing and frequency of the eruptions. In the following discussion, we do not consider the age values that are not statistically significant (reported in italics in Table 4). This is the case of the paleo-activity period, for which a reliable average recurrence time of eruption cannot be estimated. In fact, all the units dated in the time span 808-653 ka are intercalated with the fluvial-lacustrine deposits of the Paleo-Tiber River and, apart from the PG unit, are only known from borehole cores^24^. Therefore, the resulting 107 kyr-long dormancy (Table 4) likely reflects an incomplete record.

**Table 4:** Geochronologic summary of the MS activity.

ACTIVITY PHASE	UNIT	AGE ± 2σ (ka)	Ref	Inter-eruptive dormancy (kyr)	Average eruption recurrence (kyr)		Longest dormancy (kyr)
PALEO-ACTIVITY	1) PT-A	808 ± 6	^24^				107 ± 10
	2) PT-B	805 ± 7	^24^	3			
	3) PT-C	801 ± 9	^24^	4			
	4) PT-D	788 ± 13	^24^	13			
	5) PG	760 ± 6	^24^	28			
	6) VM	653 ± 4	^24^	107			
	7) SC	614 ± 4	^24^	39			
MORLUPO	8) FAD1-TGCP	589 ± 4	^24^	**25**	24.5 ± 5.6		31 ± 18
	9) FAD2	558 ± 14	^24^	31			
	10) FAD3-TGVT	546 ± 5	^24^	12			
	11) TGPP	516 ± 1	^24^	30			
	12) GRPS	510 ± 4	^24^	6			
SOUTHERN SABATINI	13) FALL A	498 ± 2	^25^	**12**	11.0 ± 5	11.5 ± 4.5	29 ± 3
	14) FALL B	490 ± 14	^24^	8			
	15) FALL C	461 ± 2	^25^	29			
	16) FALL D-TRSN	452 ± 2	^24^	9			
	17) FALL E	450 ± 7	^24^	2			
	18) FALL F	447 ± 7	^24^	3			
	19) La Rosta	438 ± 1	tw	14			
VICO	20) R94-30C	425 ± 2	3	**13**	12.3 ± 3.5		
	21) VICO α	412 ± 2	^24^	13			
	22) VICO β	403 ± 6	^24^	9			
	23) SAAS-bottom	389 ± 4	^24^	14			
	24) SAAS-top	379 ± 40	^24^	—			
BRACCIANO	25) Cornazzano lava	329 ± 4	^23^	**60** (109)^a^			**60** ± **8**
	26) Tufo di Bracciano	323 ± 2	^23^	6	4.6 ± 4.8 (9.0 ± 6.4*, 10.3 ± 4.7**)		10 ± 8
*SACROFANO*	27) M. Musino	318 ± 6	^23^	5			
BRACCIANO	28) M. R. Romana 1	317 ± 14	^23^	1			
SACROFANO	29) MRPF	312 ± 2	^23^	5			
	30) M. Aguzzo	302 ± 6	^23^	10			
BRACCIANO	31) Aguscello	298 ± 3	^23^	4			
*SACROFANO*	32) M. Cinghiale	292 ± 2	tw	6			
BRACCIANO	33) M. R. Romana 2	288 ± 6	tw	4			
SACROFANO	34) TGdS	285 ± 1	^23^	3			
BRACCIANO	35) Vigna di Valle lava	284 ± 6	^23^	1			
	36) Pizzo di Prato	251 ± 16	^23^	**33**			**33** ± **22**
*SACROFANO*	37) M. Maggiore	207 ± 7	^23^	**44**			**44** ± **23 57** ± **23**
	38) Cas. Francalancia	206 ± 9	tw	1			
LATE PHASE	39) Vigna di Valle	194 ± 7	^23^	13 (**57**)^b^			
	40) San Bernardino	≤173	^23^	21	17.1 ± 5.0		33 ± 5
	41) Lagusiello	159 ± 4	^23^	14			
	42) Cornacchia	154 ± 7	^33^	5			
	43) Prato Fontana	134 ± 33	^33^	20			
	44) Baccano lower	132 ± 2	^23^	27			
	45) Baccano main	99 ± 3	^1^	33			
	46) Stracciacappa	97 ± 4	^23^	2			
	47) Le Cese	95 ± 5	^23^	2			
	48) M. Broccoleto	93 ± 5	^23^	2			
	49) Baccano upper	91 ± 6	^23^	2			
	50) Piana dei Falliti	90 ± 12	^23^	1			
	51) Acquarella	83 ± 4	^1^	7			
	52) Martigano	70 ± 3	^1^	13			
		PRESENT			**70**		**70** ± **3**

Available eruption ages (mean values) with analytical errors at 2σ are reported in the third column, according to their progressive numbering in Fig. 6. The time interval separating two successive eruptions (inter-eruptive dormancy) is reported in the fourth column, while the average recurrence and the longest dormancy for the different activity phases are reported in the fifth and sixth column, respectively; not statistically significant age values affected by large errors are in italics.

^a^assessed without considering the activity occurred at Vico; ^b^assessed without considering the two eruptions occurred at the Sacrofano source area; *average recurrence for the eruptions occurred at Bracciano source area; **average recurrence for the eruptions occurred at Sacrofano source area. tw: this work. See text for details and discussion.

The complete MS stratigraphic succession on top of the SC unit, spanning 614 ± 4–70 ± 3 ka, has been reconstructed based on a large number of investigated exposures^1,21–24,29–31^. Therefore, the average eruption recurrence times and dormancies (periods of quiescence) during this interval can be assessed more confidently. On these grounds, here we propose a new scheme of the MS activity phases, based on the refined eruptive history, also in light of new data from the present work.

We note that the first significant dormancy (60 kyr) that largely exceeds the average eruption recurrence times is that separating the end of the early activity of the Vico volcano from the onset at Bracciano. In contrast, the dormancies separating the previously identified activity periods of Morlupo, Southern Sabatini and Vico (Table 4, bold values in fifth column) are in the same order of the average eruption recurrence times within these three periods (Table 4, sixth column). We also note that the early activity of Vico (#20–22) fills the eruptive gap separating Fall F (#18) from SAAS (#23–24) (Sant’Abbondio Ashfall Succession^24^), previously attributed to the Southern Sabatini. We suggest, instead, that SAAS represents the late stages of the early activity (Period I^40^) of the Vico volcano, which, according to its eruptive chronology and to the relatively small distance from the MS with respect to the other volcanic districts of the Roman Province, may be regarded as a satellite volcano. The short distance and SE location with respect to Vico of the type section of Sant’Abbondio^24^ support this hypothesis. In contrast, based on available thickness and clast size data, we attribute the Plinian fall of La Rosta (#19) to the central MS area and to the Southern Sabatini activity phase. Conversely, a markedly longer dormancy of 109 kyr (reported in brackets in Table 4) would have occurred at the MS if Vico is considered an independent volcanic system.

Notably, only two other significant dormancies characterized the MS eruptive activity in the last 329 kyr, i.e.: 33 kyr between the Vigna di Valle lava flow (#35) and the Pizzo di Prato eruption (#36), and 44 kyr between the latter and Monte Maggiore scoria cone activity (#37). Remarkably, the later activity at Vico (Periods II and III^40^) overlaps part of the Bracciano, Sacrofano and Late Phase activities at MS, as well as the first of these two dormancies, without affecting the second one (Fig. 7b).

Several distinct volcanic phases can be defined in the Bracciano and Sacrofano source areas. The earliest one, responsible for the formation of the Bracciano caldera, included the Tufo di Bracciano major explosive eruption (323 ± 2 ka), followed by the activity of peripheral scoria cones (e.g., Monte Rocca Romana 1, 317 ± 14 ka) and phreatomagmatic centers (e.g., Aguscello, 298 ± 3 ka), located to the NW of the caldera, and by final effusive activity (e.g., Monte Rocca Romana 2, 288 ± 6 ka and Vigna di Valle lava flow, 284 ± 6 ka).

In the overall, including the early Cornazzano lava flow, the Bracciano phase lasted 45 kyr (329 ± 2 through 284 ± 2 ka), with an average eruption recurrence of 9 kyr (Table 4). The Pizzo di Prato major explosive eruption (#36) occurred 33 kyr after this rather continuous, progressively waning phase. However, this relatively long dormancy may be an artifact due to an incomplete record of dated products and/or the large error (16 kyr) associated with this eruption age, which might reduce this dormancy up to 11 kyr (also considering the error associated with the preceding event).

In contrast to Bracciano, the eruptive events occurred in the same time span in the Sacrofano area do not define a clear trend. Specifically, scoria cone activity in this area seems unrelated, both in space and time, with the two main events of the Magliano Romano (#29) and the caldera-forming Tufo Giallo di Sacrofano (#34). By also considering the broadly coeval Monte Maggiore and Casale Francalancia (#38) eccentric scoria cones (207 ± 7 and 206 ± 9 ka, respectively) and Monte Broccoleto (94 ± 5 ka), it becomes apparent a spatial association with the main N-S fault zone (Fig. 3), which may have controlled the locations of multiple monogenetic centers in the 318-95 ka interval. The 207-206 ka (#37–38) Strombolian eruptions occurred 44 kyr after Pizzo di Prato, thus marking the second longest dormancy of the entire MS activity (Table 4), including the satellite activity of Vico (Fig. 7a).

Considering together the activities of Bracciano and Sacrofano source areas from 318 ± 6 to 284 ± 2 ka, it would result an average recurrence of 4.5 kyr, with inter-eruptive intervals ranging 1–8 kyr (Table 4). Otherwise, for the sole Sacrofano area in the same time span, an average recurrence of 10.3 kyr would result, with inter-eruptive intervals of 8–15 kyr (Table 4). This volcanic period, characterized by such high eruption frequency, parallels the prolonged Strombolian-effusive activity of the Faete Phase at CA (see Fig. 7c) in approximately the same time span (308-270 ka), correlated with a climax of tectonic activity on the local N-S faults^41^.

However, at Sacrofano, two major explosive eruptions occurred in this period, separated by 25 kyr, while enduring Strombolian-effusive activity characterized the Faete Phase until 240 ka. At CA, the shift to such low-energy style, coupled with an increase in the eruption frequency, was attributed to the mainly transpressive features of the N-S fault zone. In contrast, at MS, a right step characterizes the N-S fault zone developed through the Treja and San Martino stream valleys (Fig. 3), which, given the right-lateral movement of these faults, results into transtensive features (see^42^, for an in-depth discussion). Therefore, strike-slip tectonic activity in this area of the MS may have favored magma uprising and triggered eruptions of higher explosivity.

The above chronology highlights a 57 kyr dormancy in the Bracciano source area, before the onset of the Late Phase of activity at 194 ± 7 ka (Vigna di Valle phreatomagmatic eruption, #39). This late activity was characterized by homogeneous eruptive style, with a number of monogenetic or, in some cases, polygenetic (e.g., Baccano and Martignano) phreatomagmatic centers, with erupted volumes in the order of 0.1-1.0 km^3^ each (Fig. 7b). This activity initially took place along the eastern rim of the Bracciano caldera (i.e., Vigna di Valle, San Bernardino, Lagusello craters), and since 130 ka migrated to the east, as far as to the Baccano and Sacrofano calderas, while isolated activity occurred in the northern sector (Prato Fontana and Cornacchia lava flows, Fig. 3).

A climactic phase, from 99 ± 3 to 90 ± 12 ka, clustered at Baccano and surrounding centers, with the exception of the Monte Broccoleto scoria cone along the Sacrofano caldera rim. Besides a high-frequency climax (average 2 kyr inter-eruptive intervals; #46–50 in Table 4), an average recurrence of 17 kyr is observed for the whole Late Phase of activity, with inter-eruptive intervals ranging 5–33 kyr. However, this recurrence value is probably underestimated, since it does not consider several eruptive centers located in the northern sector, for which only one precise age constraint of 154 ± 7 ka is available so far (Fig. 3).

From a hazard perspective, the time of 70 ± 3 kyr elapsed since the last documented eruption at MS (i.e., at Martignano) seems to exceed all the previous average and longest dormancies, possibly suggesting an extinguishment of eruptive activity in the district. However, considering the uncertainties associated with the ^40^Ar/^39^Ar ages, the previously occurred longest dormancies may be even extended to 68 and 67 kyr (i.e., pre- and post-Bracciano, respectively), thus closely matching the minimum dormancy of 67 kyr since the last eruption at Martignano. Therefore, the onset of a new activity phase in the next future at the MS cannot be ruled out.

### Notes on the VU eruptive activity

A less detailed eruptive history is provided by literature data for the VU, with respect to that reconstructed here for the MS and CA. A compilation of most of the existing data was provided in^32^. A selection of these dates, excluding a set of K/Ar age determinations performed in the 80’s which have poor analytical control and reliability, has been recently compared with a record of single crystal ages from six samples of primary and reworked volcanic deposits of the Bolsena-Orvieto phase of activity^37^. In the attempt to provide insights on the whole VU eruption history, these authors proposed that several, statistically significant, crystal population ages might pertain to xenocrysts from previous eruptions. Here we included the dataset compiled in^37^ to provide a tentative reconstruction of the VU eruption history in comparison with MS and CA (Fig. 7a). Based on available data, and with caution due to their incompleteness, we remark that the dormancy period of 111 ± 18 ka since the last documented eruption largely overruns the longest quiescence ever occurred since 590 ka at the VU.

### Final remarks: state of volcanic activity in the Roman area

The new ^40^Ar/^39^Ar data for the MS and VU integrates the existing geochronologic dataset of volcanic products of the Roman Province, allowing some general inferences on the state of volcanic activity in the region. Quite remarkably, the timing of eruptive history (average eruption recurrence and dormancy periods) at CA (Fig. 7c) shows a peculiar, quasi-periodic eruptive behavior, as widely discussed in^43,44^. A relatively limited number of eruptive events occurred with regular frequency, irrespective of erupted volumes through time, allowing^43,44^ to assess a precise average eruption recurrence and dormancy period. Both these parameters resulted in the same order of the time elapsed since the onset of the last eruptive cycle at 41 ± 2 ka, and since the last eruption occurred at 36 ± 1 ka from the Albano composite maar (Fig. 7c). Moreover, the time lapse since the last eruption equals the overall average dormancy at CA during the last 100 kyr. On these grounds, a number of unrest indicators, such as active uplift in the volcanic area hosting the most recent activity, seismic swarms, gas emissions and inferences on a recent switch in the local stress field, strongly suggest that the CA is an active, quiescent, volcano (e.g.^2,43^).

Conversely, at MS, the lack of unrest indicators and the 70 kyr-long time elapsed since the last documented eruption might suggest that volcanic activity is extinguished. However, Fig. 7 highlights an overall close similarity between the MS and CA eruptive histories since 900 ka and a strict coupling in time and in eruptive magnitude between the volcanic phases of the two districts. Similarly, following a paleo-activity, two early explosive eruptions, documented at 614 ± 4 and 608 ± 2 ka at MS and CA, respectively, heralded the almost contemporaneous onset of the paroxysmal phases of Morlupo-Southern Sabatini (since 589 ± 4 ka) and Tuscolano-Artemisio (since 561 ± 1 ka). Notably, the two main eruptive events, i.e., the Tufo Rosso a Scorie Nere Sabatino at MS and the Pozzolane Rosse at CA, were almost contemporaneous at 452 ± 2 and 456 ± 5 ka, respectively.

A long dormancy separated the end of the long-lasting explosive activity occurred at MS and Vico until 400 ka from the onset at 329 ka of prolonged activities in the Bracciano and in the eastern MS sectors. This eruptive gap is the only significant dissimilarity with respect to CA, where the climactic activity phase lasted until the caldera-forming Villa Senni eruption cycle at ca. 360 ka. However, the parallelism re-established, with the start of the Faete Phase at CA at ca. 308 ka and the Sacrofano activity at MS at 312 ka, both with a possible tectonic trigger (as discussed in the previous section). Finally, the coincidence between the dormancy periods at both volcanic districts since 250 ka and the onset of the late phreatomagmatic activity phases at 200 ka is straightforward.

Based on these close similarities, such that MS and CA can be defined as twin volcanic systems for most of their documented lifetime, also in light of geophysical indicators suggesting that the CA is an active volcano, we stress that the MS should not be considered an extinct volcano. Indeed, considering that the time lapse since the last eruption is in the same order (ca. 70 kyr) of the dormancies that separated the three main phases of activity occurred in the last 600 kyr, there is no objective element allowing to exclude that a new volcanic phase might occur at MS in the near future.

On the other hand, at a broader regional scale, if we also take into account the activities at VU and Vico (although less constrained), based on the ages of the last documented eruptive events (i.e., 111 ka at VU, 95 ka at Vico^40^, 70 ka at MS^1^, and 36 ka at CA^44,45^; Fig. 7d), apparently the waning (or, possibly, cessation) of eruptive activity shifted progressively from NW to SE. Such observation might suggest a migration of the deep magma source beneath the Roman Province and that magma recharge will be eventually no more active beneath the MS in the near future.

Moreover, we notice a striking “relay behavior” of the main potassic districts of the Roman Province. The waning stage of activity at VU (150-110 ka) was concomitant to an activity climax at Vico (Vico III): including the main caldera-forming event of Tufo Rosso a Scorie Nere Vicano, Fig. 7a); then, the waning stage of activity at Vico (95 ka) was concomitant to a peak of phreatomagmatic activity at MS; again, the last documented eruption at MS (70 ka) was accompanied by the onset of Albano cycle at CA (Fig. 7c). Finally, the last eruptive phase at Albano (40-36 ka) was broadly coincident with the climactic super-eruption of the 39 ka Campanian Ignimbrite at Campi Flegrei^46^. Clearly, the focus of volcanic activity seems to be shifting through time from Northern Latium, through the Roman area, toward the Neapolitan area (Campi Flegrei-Ischia and Somma-Vesuvius^47–51^. Also, the late CA activity shows transitional geochemical features towards the Neapolitan magmatic compositions^45^ However, this general tendency does not exclude the possible occurrence in the future of eruptive codas (if not new climactic phases) from the dormant volcanoes at the gates of Rome.

## Methods

### ^40^Ar/^39^Ar dating

Fresh, unaltered, vesicle free interiors of lava samples are crushed using a tungsten carbide jaw crusher and sorted into particular size ranges by sieving. Sieved fractions were then repeatedly washed ultrasonically in DI water to remove any adhering dust, and then washed in 10% (1.2 M) HCl or 25% (3.0 M) HCl to remove minor alteration minerals that may be present. The acid leaching steps are followed by several ultrasonic rinses using DI water. The dried samples go through a magnetic separation using a Sm-Co hand magnet to remove magnetite bearing groundmass from mafic phenocrysts like olivine, pyro and plagioclase. The final processing step for a groundmass separates is hand picking under the microscope to remove any polymineral grains or any altered groundmass grains that survived the acid leaching process. Completed groundmass separate that is homogeneous and free of any of crystals.

For tephra, pyroclastic and volcaniclastic deposits, samples are crushed, sieved, and washed using the samples methods as those for the groundmass. Washed/dried samples are then passed through a Frantz barrier separator to isolate the highly purified non-magnetic crystals. For some samples that are rich in glass, quartz and feldspar, the nonmagnetic fraction following Frantz separation must then be put through a series of density separations using methylene iodide (specific gravity = 3.2 g/cm3) that is diluted to specific densities using acetone. After density separations, all minerals are ultrasonically rinsed in acetone and then mounted on glass slides so that mineral compositions can be evaluated using a scanning electron microscope (SEM).

The samples were irradiated in the CLICIT facility at the Oregon State University TRIGA reactor and were monitored with the 1.1864 Ma Alder Creek sanidine^27^. Single-crystal total fusion analyses were performed with a Photon Machines Fusions 60 W CO_2_ laser. All argon isotopic analyses were done using a Noblesse 5 collectors mass spectrometer following the procedures of^27^.

### Petrographic and SEM analysis

Textural aspects of the recovered samples were firstly analyzed in thin section at the optical microscope and successively at the Scanning Electron Microscopy (SEM), using a FEI Quanta-400 at the Earth Sciences Department (Sapienza University, Rome, Italy). Phase compositions were analyzed at the Consiglio Nazionale delle Ricerche-Istituto di Geologia Ambientale e Geoingegneria (CNR-IGAG, Rome), by a Cameca SX50 electron microprobe equipped with five wavelength dispersive spectrometers (WDS).

### A-InSAR analysis

A-InSAR processing for the descending and the ascending orbits were repeated for two different areas, resulting in a total of four processing: i) a larger area, extending from the Tyrrhenian coast to the Tiber valley and comprising the sector affected by differential uplift since 125 ka identified in a previous study^1^; a smaller area corresponding to the MS. The retrieved results were uploaded into a dedicated GIS, in order to perform an accurate analysis and comparison among the various outcomes.

InSAR analyses were performed using the multitemporal differential SAR interferometry (Advanced InSAR) technique known as Persistent Scatterers (PS^51^). This methodology allows to detect ground displacements with millimeter accuracy by analyzing SAR image stacks, providing the mean ground velocities and the relative displacement time series along the sensor line of sight (sensor-target direction, LOS) for the coherent targets.

We used images acquired from the Sentinel-1A/B satellites and operated by the European Space Agency (ESA) with a revisiting time over the investigated area of 6 days. The covered temporal period spans from September 2016 to March 2018 for the descending orbit (84 images) and from January 2016 to January 2018 along the ascending orbit (99 images). The digital elevation model (DEM) adopted for the differential interferograms generation (removal of the topographic contribution) was the SRTM-1 (30 meters) in the WGS84 projection.

A series of differential interferograms with respect to a single master were generated by the adopted algorithm, aimed to initially select persistent scatterer candidates (PSC). The time series of the amplitude values of each pixel were analyzed in order to identify stable targets in the area of interest. Only pixels exhibiting a very “*stable*” sequence of amplitude values were selected to be used as targets affected by small geometrical and temporal decorrelation. On the PSC sparse grid, we estimated and removed the digital elevation model (DEM) errors, the line of sight (LOS) velocities, and the linear atmospheric phase (APS) contributions. The APS contributions were estimated and resampled on the uniform image grid by interpolation. All differential interferograms were then compensated for the estimated atmospheric contribution. A joint estimation of both DEM errors and target velocity was then carried out on a pixel-by-pixel basis to obtain the optimized PS velocity estimation.

Note that, due to the different angle of view (Line of Sight) of each orbit, the agreement in terms of sign between the retrieved deformation patterns on both ascending and descending orbits indicates a dominant vertical component of the movement (i.e., uplift for positive values and subsidence in case of negative values). In contrast, the inversion of the retrieved velocities sign reveals a prevalent horizontal component of movement^52^.

## Supplementary information

Supplementary Material 1.

Supplementary Material 2A.

Supplementary Material 2B.

Supplementary Material 2C.

Supplementary Material 3.

## Data Availability

All data generated or analysed during this study are included in this published article.
