# Supplementary material for: Monti Sabatini and Colli Albani: the dormant twin volcanoes at the gates of Rome
Source: Sci Rep. 2020 May 26;10:8666. doi: 10.1038/s41598-020-65394-2 (PMC7251092; doi:10.1038/s41598-020-65394-2)
Supplement: Supplementary file 1 — Supplementary Material 1. [file 41598_2020_65394_MOESM1_ESM.doc]

**Monti Sabatini and Colli Albani: the dormant twin volcanoes at the gates of Rome**

Marra, F.^1^, Castellano, C.^1^, Cucci, L.^1^, Florindo, F.^1^, Gaeta, M.^2^, Jicha, B.^3^, Palladino, D.M.^2^, Sottili, G.^2^, Tertulliani, A.^1^ ,Tolomei, C.^1^

1) Istituto Nazionale di Geofisica e Vulcanologia, Via di Vigna Murata 605, 00143 Rome, Italy

2) Dipartimento di Scienze della Terra, “Sapienza” Università di Roma, Piazzale Aldo Moro 5, 00185 Roma, Italy

3) Department of Geoscience, University of Wisconsin-Madison, USA

*Corresponding author: fabrizio.marra@ingv.it

**Supplementary Material # 1 - Phases of activity at the Monti Sabatini Volcanic District**

**Paleo-activity (808±6 - 614±3 ka)**

A poorly known Paleo-activity consists in several tephra layers intercalated within fluvial deposits of the Paleo-Tiber River, dated between 808±6 ka and 611±6 ka [1, 2, 3]. The source areas of these eruptions are uncertain.

**Morlupo activity (589±4 - 510±4 ka)**

It took place in the eastern sector of the MSVD and comprised three main subphases [4, 5] named after the principal pyroclastic-flow units emplaced during the eruptive climax. It includes the Tufo Giallo di Castelnuovo di Porto Succession (TGCP, 589±4 ka), the Tufo Giallo della Via Tiberina succession (TGVT, 546±3), and the Tufo Giallo di Prima Porta succession (TGPP, 516±1) - Grottarossa Pyroclastic Succesion (GRPS, 510 ±4 ka). Three Plinian fallouts (FADs) are also associated with this phase.

**The Southern Sabatini phase (498±2 - 389±4 ka)**

This intense eruptive phase developed in the central-southern sector of the MSVD (Fig. 3) and marked the climax of its activity history in terms of eruption magnitudes [6]. The deposits from the main eruptions are the Tufi Terrosi con Pomici Bianche Plinian fallouts (498±2 - 461±2 ka) and the Tufo Rosso a Scorie Nere (TRSN) Eruption Cycle (452±2 - 447±7 ka) [5].

The post-climactic Southern Sabatini explosive activity, informally named San Abbondio Ash-lapilli Succession (SAAS, 389±4 ka), is represented by a ~10 m-thick pyroclastic succession that covers two Plinian fall deposits from the Vico Volcanic District (Vico α dated at 412±7-419±6 and Vico β dated at 403±6 ka; [7] and topped by the 312±2 ka Magliano Romano Plinian fall deposit [8]. The SAAS has poor upper geochronologic constraints represented by an age of 379±40 ka and is the only intervening volcanic activity so far recognized at the MSVD in the time interval 447 - 329 ka, which marks a significant relative dormancy of 118 kyr.

In the present study we suggest that the SAAS was actually an eruption phase occurred at Vico Volcano.

**The Bracciano Caldera phase (323±2 - 194±7 ka)**

This phase, nearly contemporaneous with the Sacrofano caldera activity, is associated with the formation of the main MSVD volcano-tectonic depression (approximately ~10 km in diameter), which is today partly occupied by the Bracciano Lake [4] (Fig. 3 ). The large Tufo di Bracciano pyroclastic-flow deposit (323±2 ka, re-calculated here according to the age of 1.184 Ma for the Alder Creek Tuff sanidine from [9] was erupted during the main caldera forming event, followed by dominantly Strombolian/effusive and subordinately hydromagmatic eruptions from either scattered or clustered monogenetic centers aligned along the ring fault systems bordering the northern area of the Bracciano lake [10].

Associated with two more, late caldera-forming events are the Tufo di Pizzo Prato Unit (251±16 ka) and the Tufo di Vigna di Valle Unit (194±7 ka), cropping out along the external southwestern rim of the Bracciano lake. The hydromagmaric center of San Bernardino (≤173 ka) is also associated with the Bracciano phase [8].

**The Sacrofano Caldera phase (318±6 - 208±7 ka)**

A subdued caldera morphology is associated with the Tufo Giallo di Sacrofano Unit (285±2 ka) [4], a widespread pyroclastic-flow deposit which covers an area of some hundred square kilometers around the caldera including the north-eastern part of the Rome urban area [2]. However, the oldest product associated with this phase, characterized by strombolian and hydromagmatic activity and subordinate Plinian to sub-Plinian events, is the Monte Musino scoria cone (318±6 ka), located along the south-eastern rim of the Sacrofano caldera, and associated with a small lava flow. Stratigraphic relationships of this edifice with the large Magliano Romano Plinian fall deposit (312±2 ka), cropping out extensively in the north-eastern sector of the MSVD, are unclear. Moreover, to the Sacrofano Caldera phase are associated the Monte Aguzzo Scoria cone (302±6 ka), located ~4 km to the southern rim of the Sacrofano caldera, and the much more later Monte Maggiore scoria cone (208±7 ka) located on the northern rim (Fig. 3).

**The Late activity (~160 – 70±3 ka)**

The last and the most recent MSVD activity was characterized by minor volume hydromagmatic, strombolian and effusive eruptions through several scattered or clustered monogenetic centers around the Bracciano and Sacrofano calderas. A second vent area active in the interval 150-130 ka [11] was that around the Monterosi hydromagmartic crater, located in the northern sector of the MSVD (Figure 3).

In the northern area are recognized: the Cornacchia Lava (154±7 ka [11]) directly overlying the Tufo Rosso a Scorie Nere Vicano unit (TRSNV, 150±4 ka, [12]); the Lagusiello Maar succession (159±4 ka); and the Prato Fontana lava (134±33 ka), which crops out in the northern MSVD, near the Monterosi Lake. Attempting at dating the activity of Monterosi produced a stratigraphically inconsistent age 259 ka (youngest crystal), conflicting with evidence of lithic inclusions of TRSNV [8]. To this area may be related also the activity of the San Bernardino center, located on the northern rim of Bracciano Lake, which has poor geochronologic constraints represented by a youngest crystal age, providing a terminus post-quem ≤173±5 ka.

In the central area are recognized the Baccano Lower, Baccano Main and Baccano top units (132±2, 99±3 ka, 92±6 ka), constituted by complex pyroclastic successions marked by multiple erosional unconformities and paleosoils related to a composite maar-caldera system [10, 13,14]; the Stracciacappa Maar Unit (98±4 ka) represents the youngest eruptive unit from a polygenetic maar [15]; the small Le Cese hydromagmatic center (96±5 ka); the Monte Broccoleto scoria cone (94±5 ka), located at South East of the Sacrofano caldera, consists of a poorly consolidated strombolian fallout and a small lava flow; the Piana dei Falliti maar (90±12 ka); the Martignano polygenetic maar (Martignano Lower unit, 87±5 ka, Martignano Upper Unit, 70±3 ka). The Martignano maar displays composite volcanic morphologies with at least three coalescing craters, overall 2.5 km across [10].

REFERENCES

[1] Karner, D.B. & Renne, P.R. 40Ar/39Ar geochronology of Roman Volcanic Province tephra in the Tiber River Valley: Age calibration of middle Pleistocene sea-level changes. *Geological Society of America Bulletin* **110**, 740-747 (1998).

[2] Karner, D. B., Marra, F. & Renne P. R. The history of the Monti Sabatini and Alban Hills volcanoes: groundwork for assessing volcanic-tectonic hazards for Rome. *J. Volcanol. Geotherm. Res.* **107**, 185-219 (2001).

[3] Florindo, F., et al. Radioisotopic age constraints for Glacial Terminations IX and VII from aggradational sections of the Tiber River delta in Rome, Italy. *Earth Planet. Sci. Lett.* **256**, 61-80 (2007). doi: 10.1016/j.epsl.2007.01.014.

[4] De Rita, D., Funiciello, R., Corda, L., Sposato, A. & Rossi, U. Volcanic Units. In: Di Filippo, M., (Ed.), Sabatini Volcanic Complex, *Quad. Ric. Sci.* 114, Progetto Finalizzato Geodinamica C.N.R., Roma, 33-79 (1993).

[5] Marra, F., *et al*. Major explosive activity in the Sabatini Volcanic District (central Italy) over the 800-390 ka interval: geochronological - geochemical overview and tephrostratigraphic implications, *Quaternary Science Reviews* **94**, 74-101 (2014).

[6] Sottili, G., Palladino, D.M. & Zanon, V. Plinian activity during the early eruptive history of the Sabatini Volcanic District, Central Italy. *J. Volcanol. Geotherm. Res.* 135, 361-379 (2004).

[7] Barberi, F., et al. Plio-Pleistocene geological evolution of the geothermal area of Tuscany and Latium. *Mem. Descr. Carta Geol. d' It.* **49**, 77-134 (1994).

[4] Peccerillo, A. *Plio-Quaternary volcanism in Italy. Petrology, Geochemistry, Geodynamics*. Springer, Heidelberg (2005).

[8] Sottili, G. *et al.* Geochronology of the most recent activity in the Sabatini Volcanic District, Roman Province, central Italy. ***J. Volcanol. Geotherm. Res.***  **196**, 20-30 (2010).

[9] Pereira, A. *et al.* 40Ar/39Ar and ESR/U‑series data for the La Polledrara di Cecanibbio archaeological site (Lazio, Italy). *Journal of Archaeological Science: Reports* **15**, 20-29 (2017). DOI:[10.1016/j.jasrep.2017.05.025](https://www.researchgate.net/deref/http%3A%2F%2Fdx.doi.org%2F10.1016%2Fj.jasrep.2017.05.025?_sg%5B0%5D=9dngrXzsnDGYGPcZqcBoGv6bPs2q1Wfllhw3l_rZT_qkhm3kXqapK1dKwXlyqJf5AyztqjU-4KDDWzRJXqxWC6t5GA.O3txQS_kbJJVn-Q5VWRtvkhdXfpdqLED8jud3E05AUAn50Pz-q_-od8Y6Suh4VN89AbxHxnNzwSOSA2pfk5TIA)

[10] Sottili, G., Palladino, D.M., Gaeta, M. & Masotta, M. Origins and energetics of maar volcanoes: examples from the ultrapotassic Sabatini Volcanic District (Roman Province, Central Italy). *Bull. Volcanol.* **74**, 163–186 (2012).

DOI 10.1007/s00445-011-0506-8

[11] Nappi, G. & Mattioli, M. Evolution of the Sabatinian Volcanic District (central Italy) as inferred by stratigraphic successions of its northern sector and geochronological data. *Per. Mineral.* **72**, 79–102 (2003).

[12] Laurenzi, M.A., & Villa, I.M. 40Ar/39Ar chronostratigraphy of the Vico ignimbrites. *Per. Mineral.* **56**, 285–293 (1987).

[13] De Rita, D., Funiciello, R., Sposato, A. & Rossi, U. Structure and evolution of the Sacrofano-Baccano caldera, Sabatini volcanic complex, Rome. *Journal of Volcanology and Geothermal Research* **17**(1-4), 219-236 (1983). DOI:[10.1016/0377-0273(83)90069-0](https://www.researchgate.net/deref/http%3A%2F%2Fdx.doi.org%2F10.1016%2F0377-0273(83)90069-0?_sg%5B0%5D=OSP4F1Z_QkE_Th4cOBJcctUGUm__9CraQVl0THvjFI8ghxZpt_GKq0h9zx-xq2nJOkej7j3pqmOh6YsiSraXxTK-TQ.dNsJM78co-2ucPFBGZAueu0f5nAjSttNYWMpxvuw2x1sj_AIW1xGm_brHuKVE6oOss-n_Ao1nj5GXhh_M0BtyQ)

[14] Buttinelli, M., De Rita, D., Cremisini, P. & Cimarelli, C. Deep explosive focal depths during maar forming magmatic-hydrothermal eruption: Baccano Crater, Central Italy. *Bull. Volcanol.* **73**(7), 899-915 (2011). DOI:10.1007/s00445-011-0466-z

[15] Valentine, G.A., Sottili, G., Palladino, D.M. & Taddeucci J. Tephra ring interpretation in light of evolving maar-diatreme concepts: Stracciacappa maar (central Italy), *J. Volcanol. Geotherm. Res.* **308**, 19-29 (2015). DOI:[10.1016/j.jvolgeores.2015.10.010](http://dx.doi.org/10.1016/j.jvolgeores.2015.10.010)
