# Supplementary material for: Monti Sabatini and Colli Albani: the dormant twin volcanoes at the gates of Rome
Source: Sci Rep. 2020 May 26;10:8666. doi: 10.1038/s41598-020-65394-2 (PMC7251092; doi:10.1038/s41598-020-65394-2)
Supplement: Supplementary file 4 — Supplementary Material 2C. [file 41598_2020_65394_MOESM4_ESM.pdf]

Monti Sabatini and Colli Albani: the dormant twin volcanoes at the gates of Rome

Marra, F.[1], Castellano, C.[1], Cucci, L.[1], Florindo, F.[1], Gaeta, M.[2], Jicha, B.[3], Palladino, D.M.[2], Sottili, G.[2], Tertulliani, A.[1] ,Tolomei, C.[1]

1) Istituto Nazionale di Geofisica e Vulcanologia, Via di Vigna Murata 605, 00143 Rome, Italy
2) Dipartimento di Scienze della Terra, "Sapienza" Università di Roma, Piazzale Aldo Moro 5, 00185 Roma, Italy
3) Department of Geoscience, University of Wisconsin-Madison, USA

*Corresponding author: fabrizio.marra@ingv.it

Supplementary Material # 2c - $^{40}Ar/^{39}Ar$ data plots

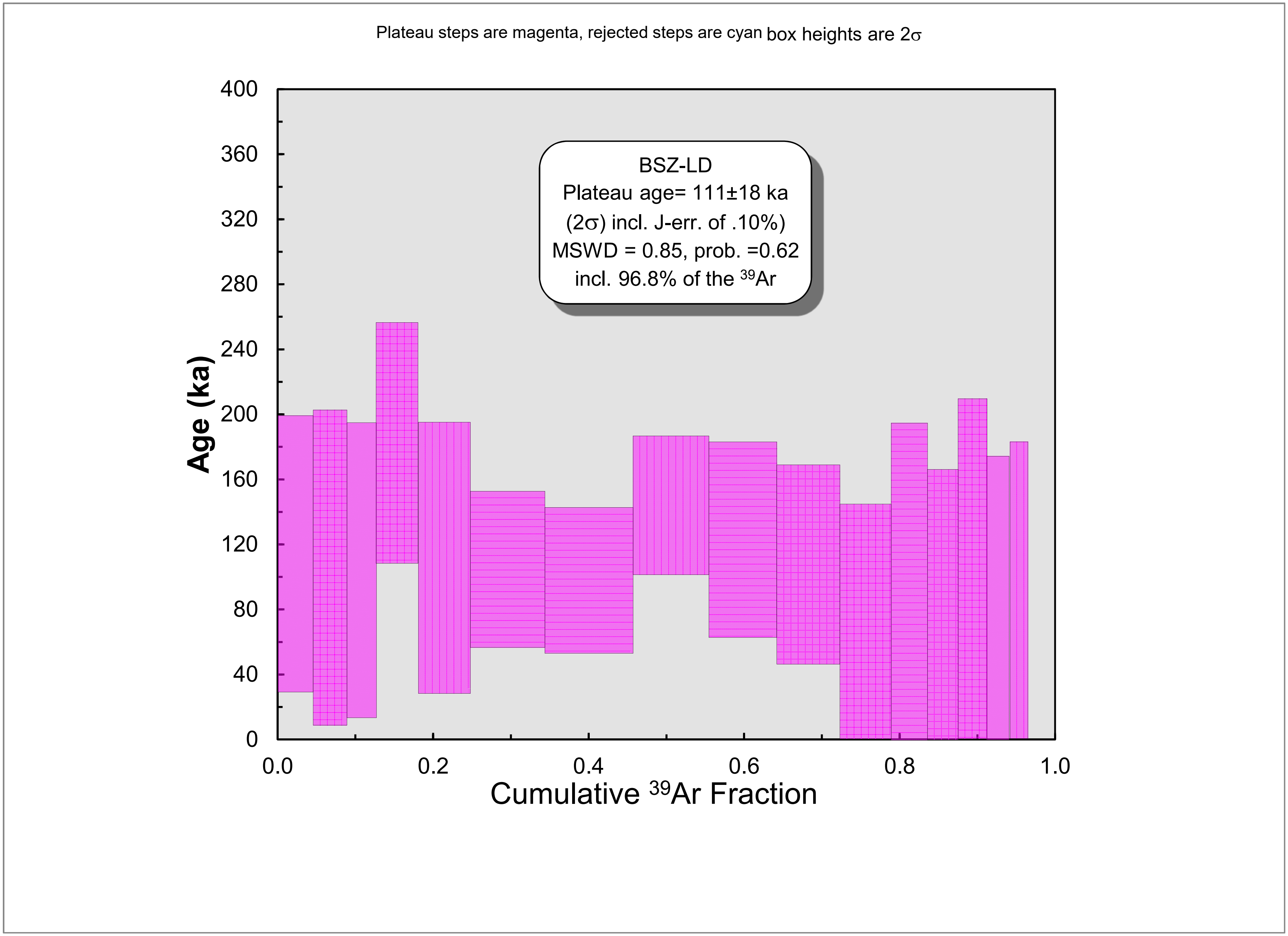
Plateau steps are magenta, rejected steps are cyan box heights are 2σ
BSZ-LD
Plateau age= 111±18 ka
(2σ) incl. J-err. of .10%)
MSWD = 0.85, prob. =0.62
incl. 96.8% of the 39Ar
Age (ka)
0
40
80
120
160
200
240
280
320
360
400
0.0
0.2
0.4
0.6
0.8
1.0
Cumulative 39Ar Fraction

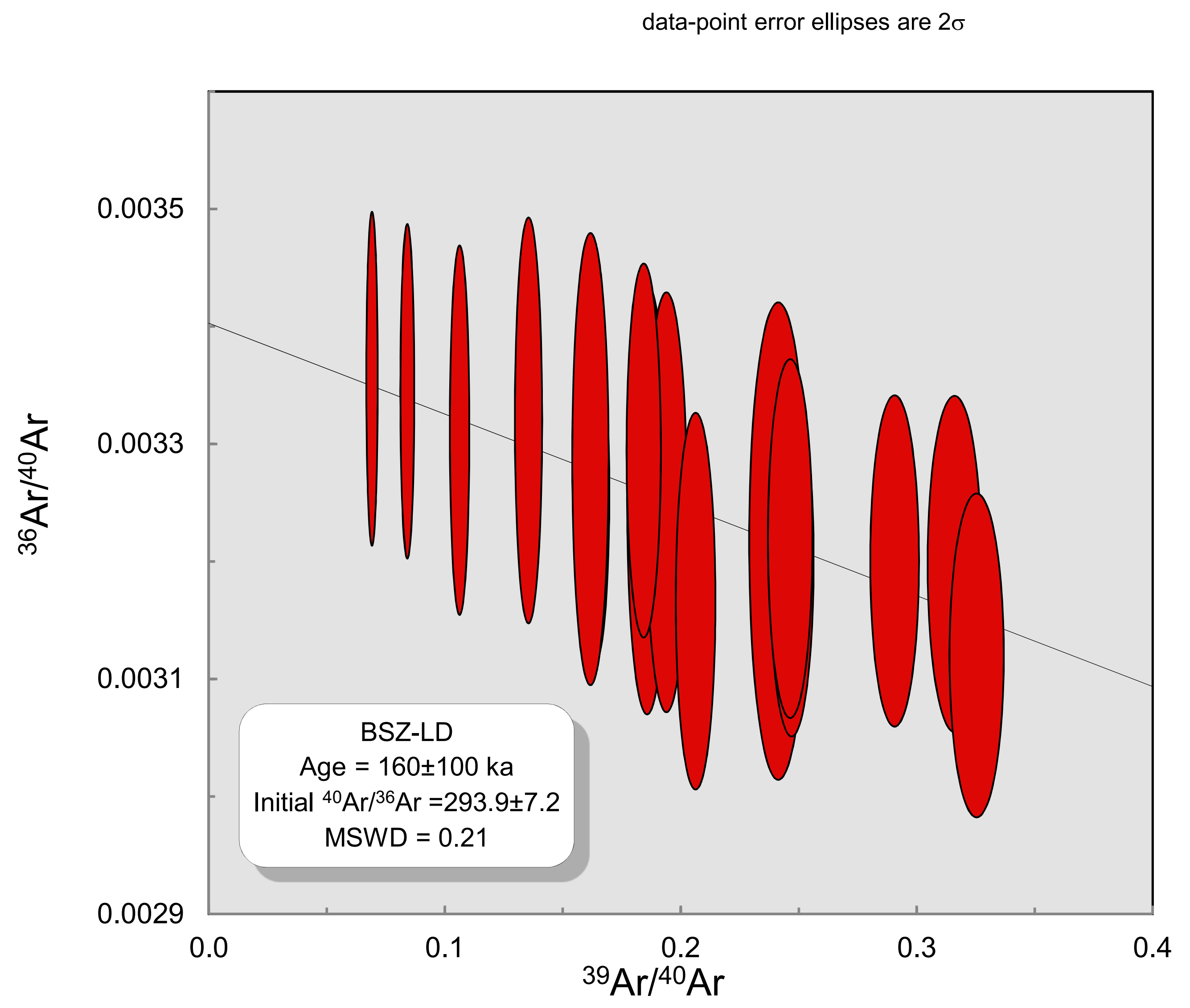

data-point error ellipses are 2σ
0.0035
0.0033
0.0031
0.0029
36Ar/40Ar
0.0
0.1
0.2
0.3
0.4
39Ar/40Ar
BSZ-LD
Age = 160±100 ka
Initial 40Ar/36Ar =293.9±7.2
MSWD = 0.21

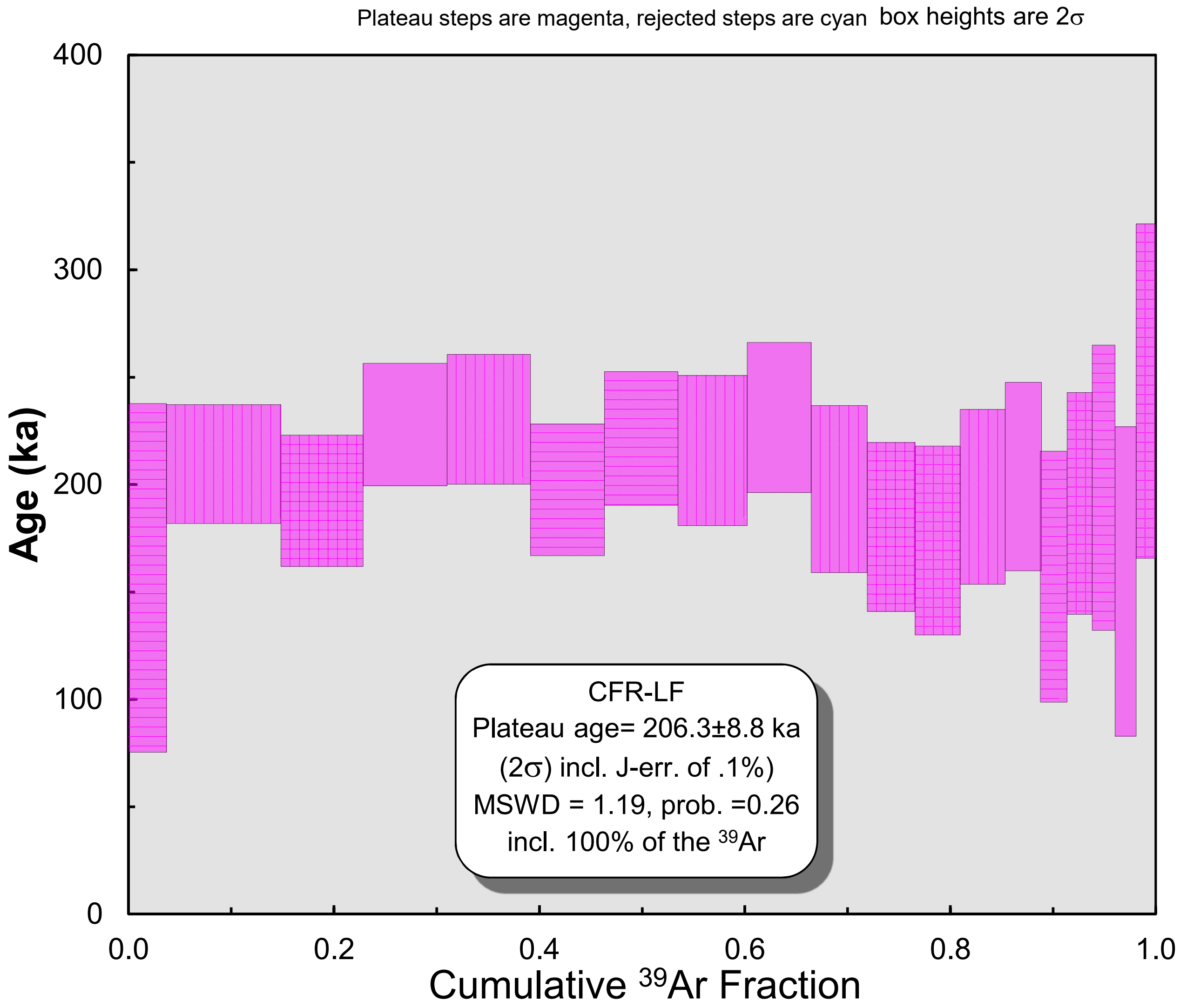
Plateau steps are magenta, rejected steps are cyan box heights are 2σ
Age (ka)
400
300
200
100
0
0.0
0.2
0.4
0.6
0.8
1.0
Cumulative 39Ar Fraction
CFR-LF
Plateau age= 206.3±8.8 ka
(2σ) incl. J-err. of .1%)
MSWD = 1.19, prob. =0.26
incl. 100% of the 39Ar

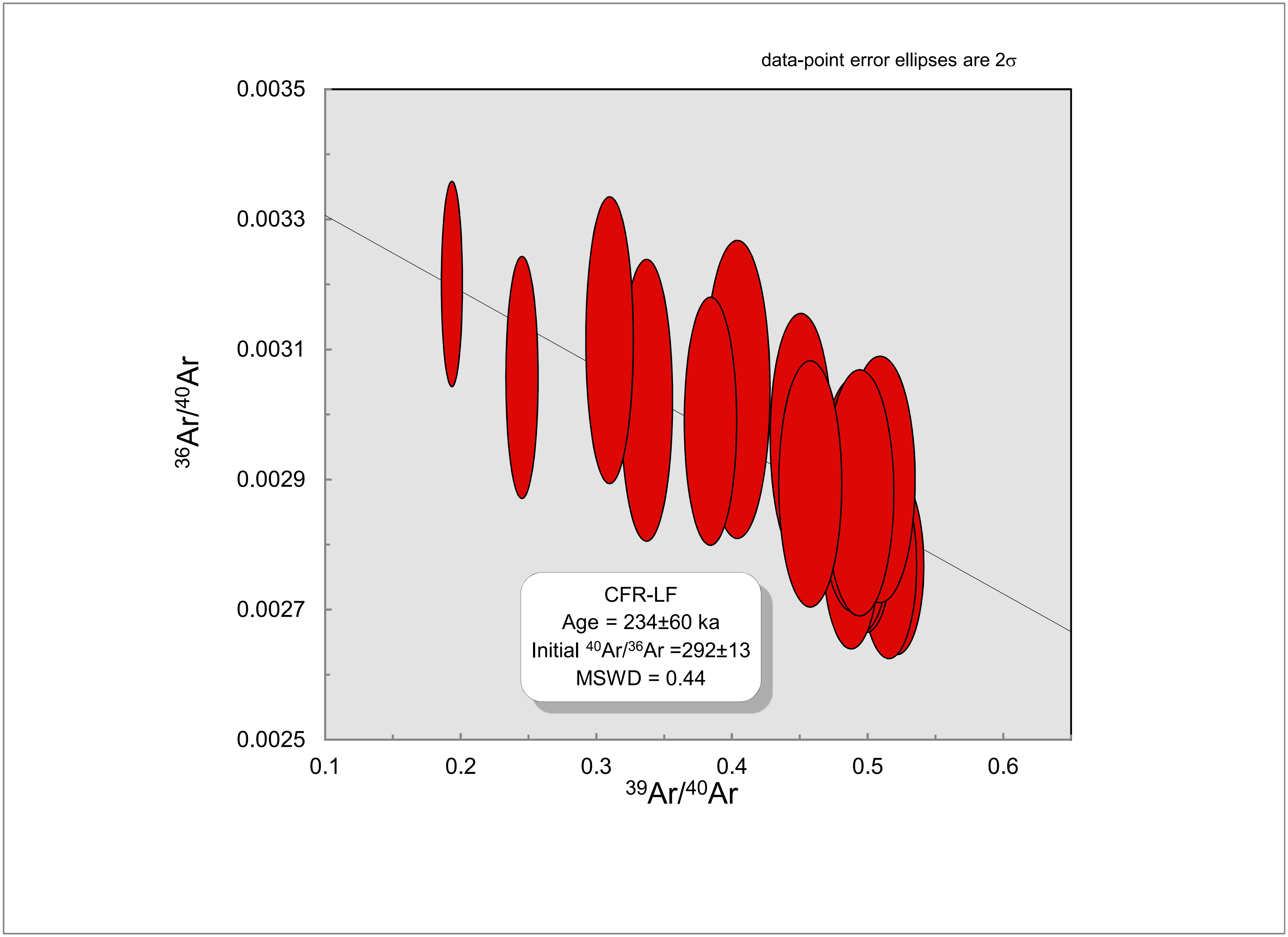

data-point error ellipses are 2σ
0.0035
0.0033
0.0031
0.0029
0.0027
0.0025
0.1
0.2
0.3
0.4
0.5
0.6
$^{36}Ar/^{40}Ar$
$^{39}Ar/^{40}Ar$
CFR-LF
Age = 234±60 ka
Initial $^{40}Ar/^{36}Ar$ =292±13
MSWD = 0.44

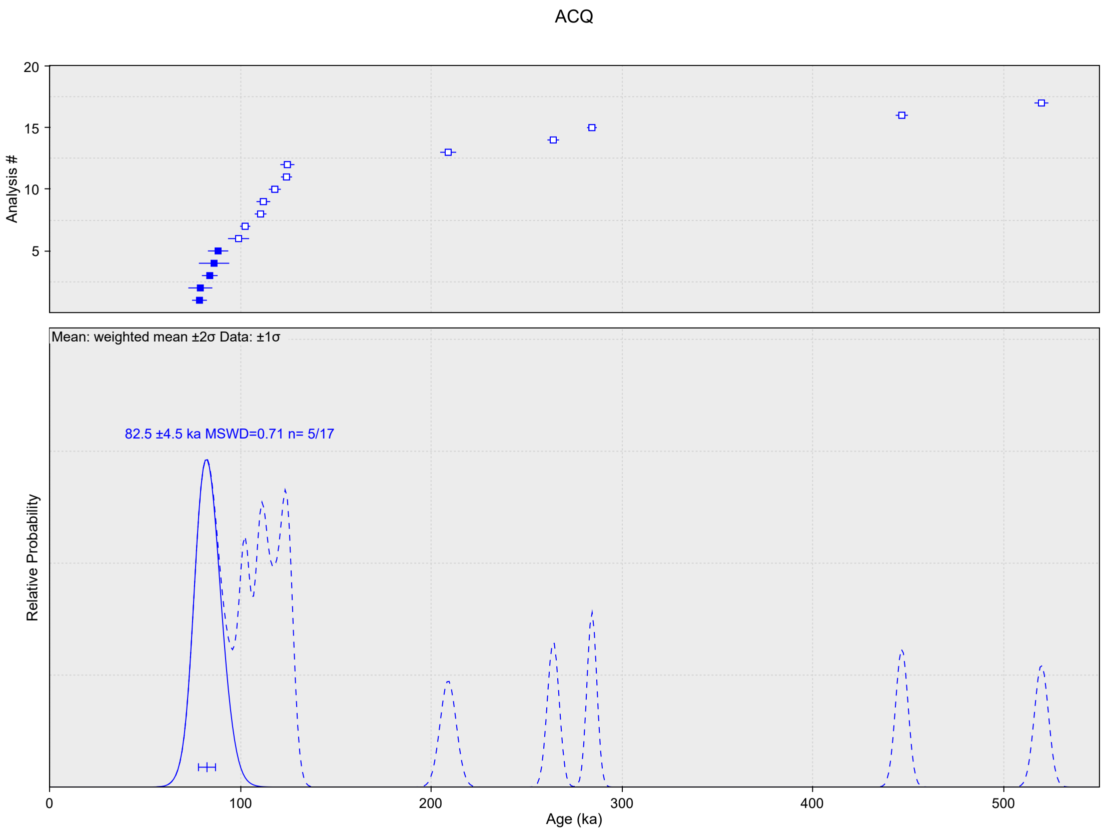
ACQ
Analysis #
Mean: weighted mean ±2σ Data: ±1σ
82.5 ±4.5 ka MSWD=0.71 n= 5/17
Relative Probability
Age (ka)

MAR-3

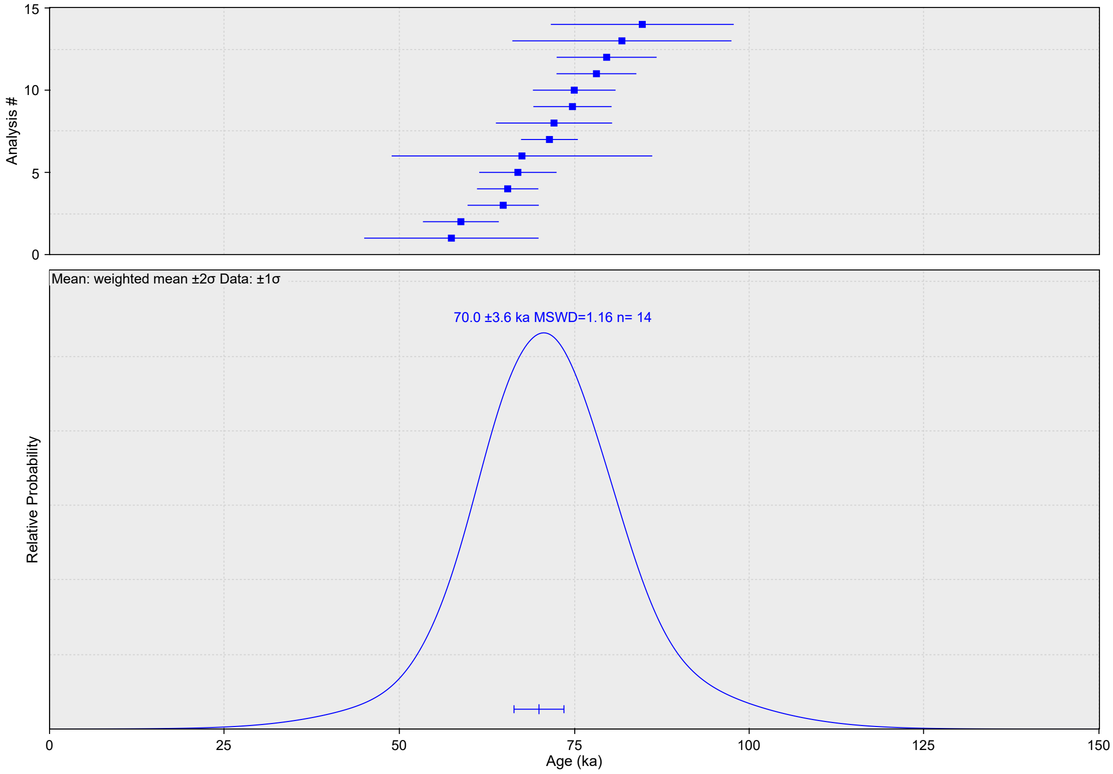

Mean: weighted mean ±2σ Data: ±1σ
70.0 ±3.6 ka MSWD=1.16 n= 14
Analysis #
Relative Probability
Age (ka)
0
5
10
15
0
25
50
75
100
125
150

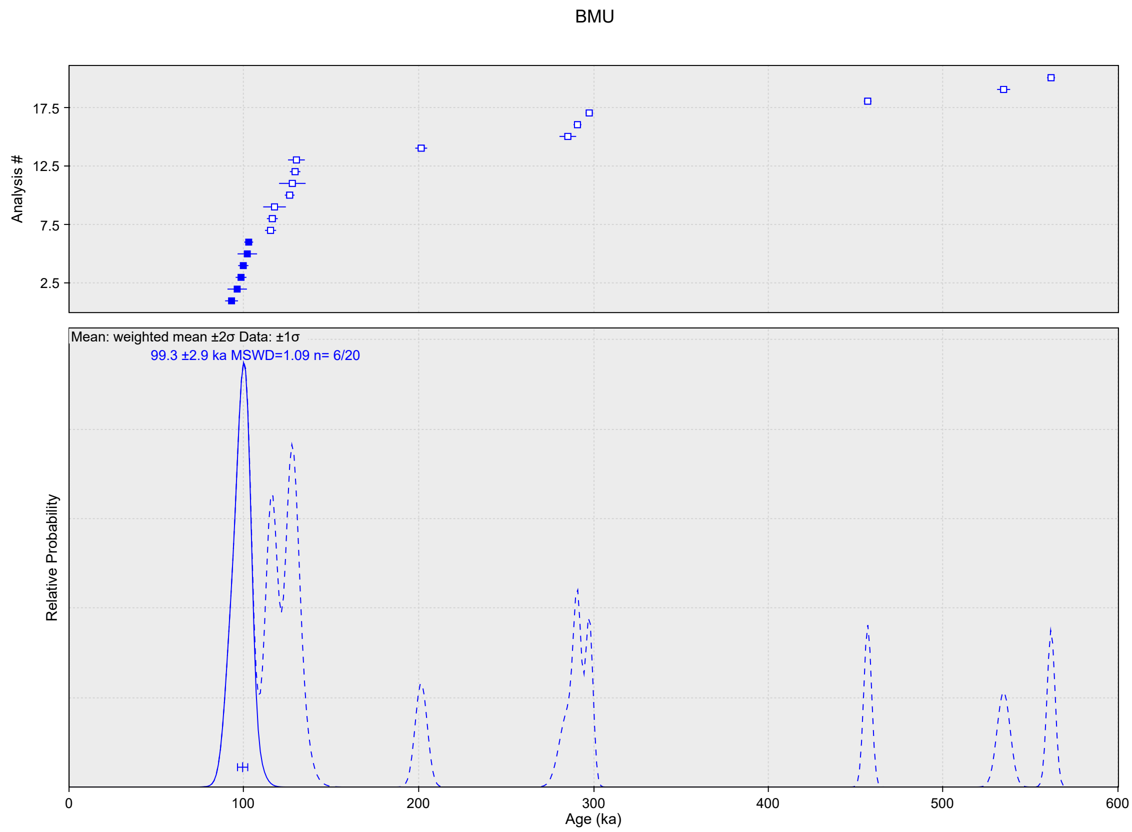

BMU
Analysis #
17.5
12.5
7.5
2.5
Mean: weighted mean ±2σ Data: ±1σ
99.3 ±2.9 ka MSWD=1.09 n= 6/20
Relative Probability
0
100
200
300
400
500
600
Age (ka)

LGC-2

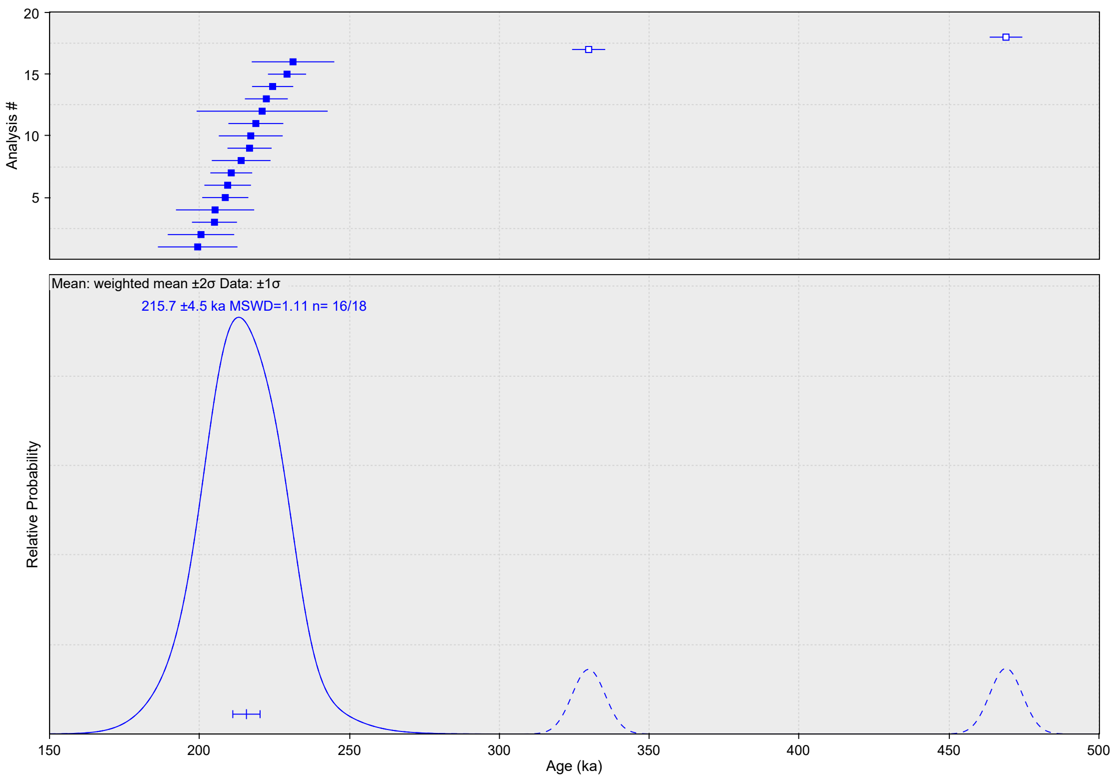

Analysis #
20
15
10
5
Mean: weighted mean ±2σ Data: ±1σ
215.7 ±4.5 ka MSWD=1.11 n= 16/18
Relative Probability
150
200
250
300
350
400
450
500
Age (ka)

VSM

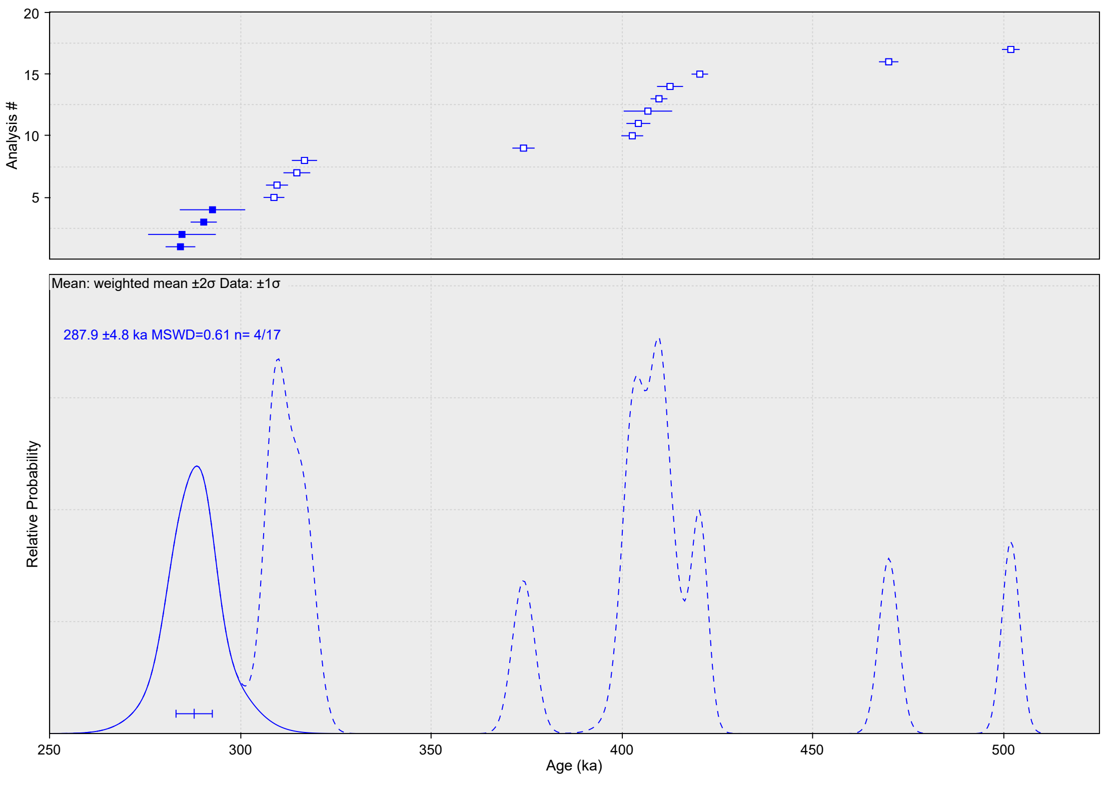

Analysis #
20
15
10
5
Mean: weighted mean ±2σ Data: ±1σ
287.9 ±4.8 ka MSWD=0.61 n= 4/17
Relative Probability
250
300
350
400
450
500
Age (ka)

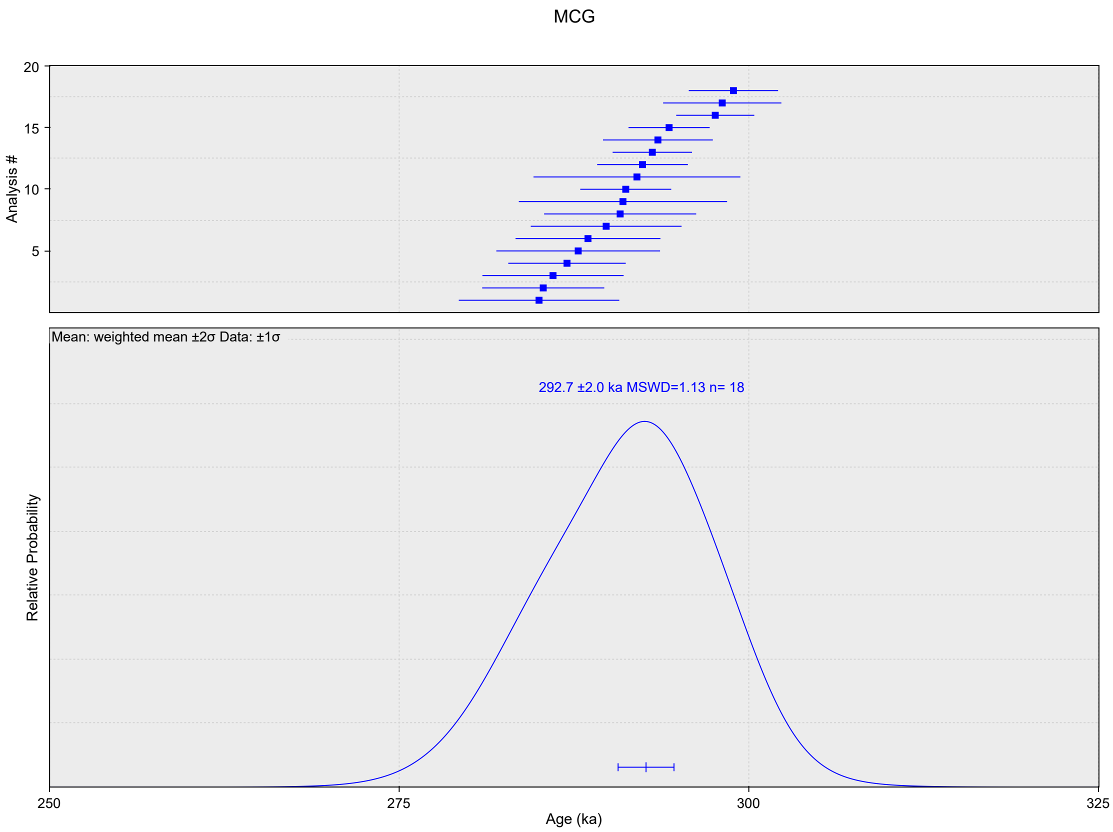
MCG
20
15
10
5
Analysis #
Mean: weighted mean ±2σ Data: ±1σ
292.7 ±2.0 ka MSWD=1.13 n= 18
Relative Probability
250
275
300
325
Age (ka)

MRR

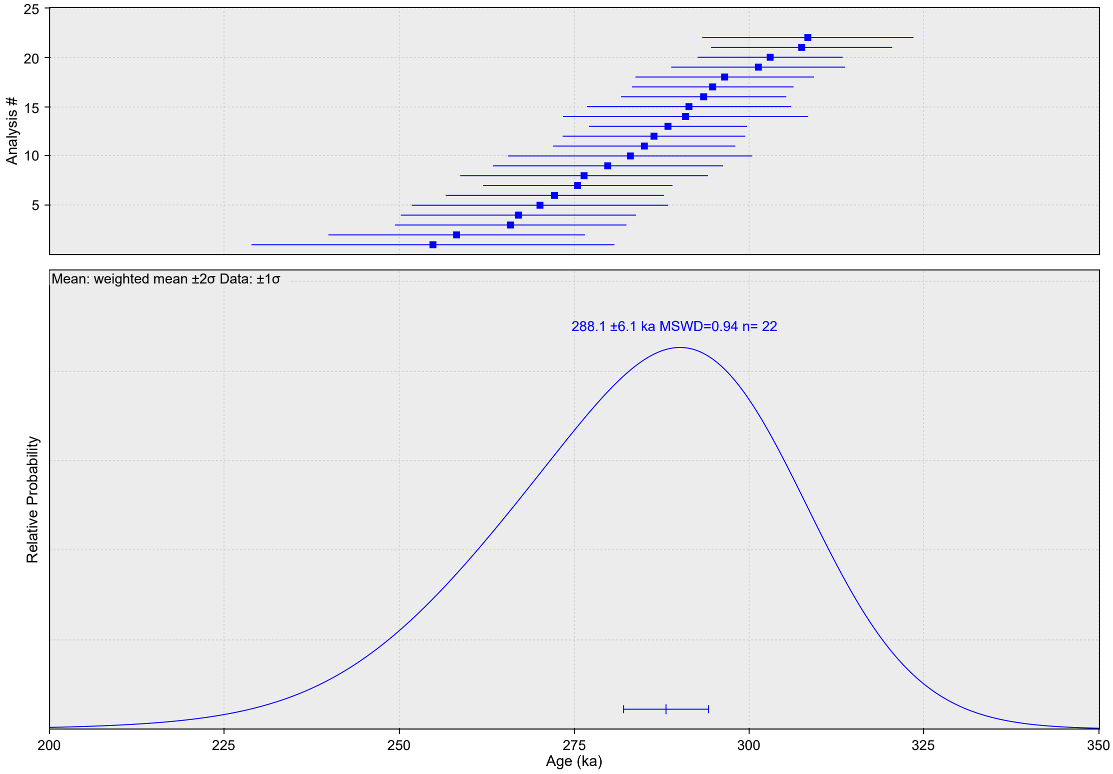

Analysis #
25
20
15
10
5
Mean: weighted mean ±2σ Data: ±1σ
288.1 ±6.1 ka MSWD=0.94 n= 22
Relative Probability
200
225
250
275
300
325
350
Age (ka)

LRS

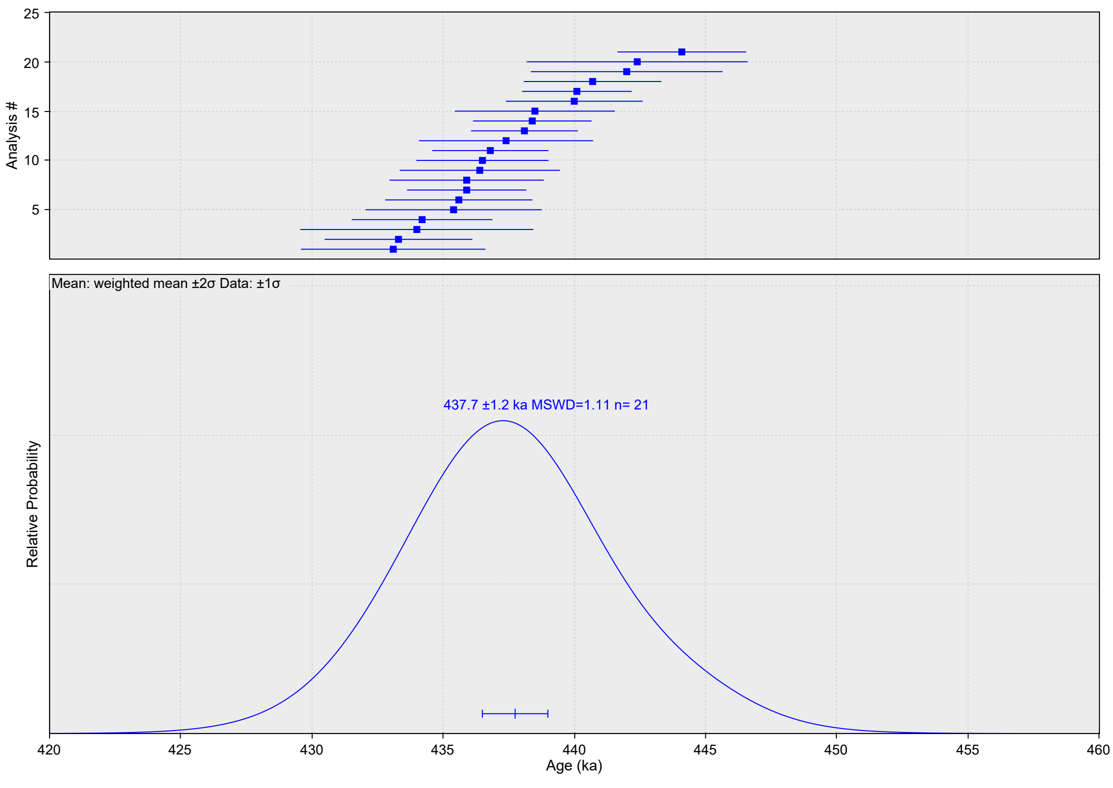

25
20
15
10
5
Analysis #
Mean: weighted mean ±2σ Data: ±1σ
437.7 ±1.2 ka MSWD=1.11 n= 21
Relative Probability
420
425
430
435
440
445
450
455
460
Age (ka)
