# Supplementary material for: Monti Sabatini and Colli Albani: the dormant twin volcanoes at the gates of Rome
Source: Sci Rep. 2020 May 26;10:8666. doi: 10.1038/s41598-020-65394-2 (PMC7251092; doi:10.1038/s41598-020-65394-2)
Supplement: Supplementary file 5 — Supplementary Material 3. [file 41598_2020_65394_MOESM5_ESM.doc]

**Monti Sabatini and Colli Albani: the dormant twin volcanoes at the gates of Rome**

Marra, F.^1^, Castellano, C.^1^, Cucci, L.^1^, Florindo, F.^1^, Gaeta, M.^2^, Jicha, B.^3^, Palladino, D.M.^2^, Sottili, G.^2^, Tertulliani, A.^1^ ,Tolomei, C.^1^

1) Istituto Nazionale di Geofisica e Vulcanologia, Via di Vigna Murata 605, 00143 Rome, Italy

2) Dipartimento di Scienze della Terra, “Sapienza” Università di Roma, Piazzale Aldo Moro 5, 00185 Roma, Italy

3) Department of Geoscience, University of Wisconsin-Madison, USA

*Corresponding author: fabrizio.marra@ingv.it

**Supplementary Material # 3 - Sample description**

**MONTI SABATINI**

**Martignano 3 (MAR-3)**

The Martignano polygenetic maar (crater size of ~2.5 km across), located to the East of Lake Bracciano (Figure 3 in main text), was formed through three main evolutionary stages, as recorded by two immature paleosoils occurring in the maar stratigraphy. The youngest Martignano pyroclastic unit, informally named Martignano 3 Unit, consists of a few tens of meter-thick, massive to weakly laminated ash deposits from pyroclastic surge activity, also including ballistic lava blocks and boulders. Along the eastern crater rim, the Martignano 3 Unit overlies the Baccano Main Eruptive Unit (see below). A previous attempt to determine the age of the youngest Martignano Unit gave a terminus post-quem age of 87±5 ka, as inferred from the youngest crystal [1]. Here, we analysed leucite crystals from juvenile grey-greenish scoria lapilli, sampled along the SW crater wall, from the Martignano 3 Unit.

**Acquarello layer (ACQ-1)**

Sample ACQ-1 refers to a ~30 cm thick fine ash layer with scarce accretionary lapilli at the middle-bottom, exposed near the Acquarello maar on the NE shore of Bracciano Lake (Figure 3 in main text). This layer appears as the topmost pyroclastic deposit in the area NE of Lake Bracciano, although a well-constrained correlation to specific events still lacks.

**Baccano Main Unit (BMU)**

The Main Baccano pyroclastic unit represents the youngest and most important pyroclastic deposit, in terms of erupted magma volume, from the polygenetic Baccano caldera, a depression ca. 3.8 km long axis in a N–S direction which developed between the major Bracciano and Sacrofano calderas (Figure 3 in main text). We collected whitish vesicular pumice lapilli from the meter-thick, massive, highly consolidated, lithic-rich, greyish ash deposit exposed on top of an erosional unconformity cutting through the underlying pyroclastic succession along the well preserved western caldera rim. Along the southern caldera rim, the Baccano Main Unit underlies a well-developed, dark brown paleosoil topped by a light grey, consolidated and cross-laminated, decimeter-thick ash deposit dated 92±6 ka (SAB-56 sample in [1]).

**Valle Santa Maria (VSM)**

The Valle Santa Maria area (Figure 3) is characterized by a series of small, coalescent maars aligned along regional tectonic fractures, likely connected with shallow aquifers [2, 3]. Previous studies suggested that the Valle Santa Maria products overlie the Tufo Rosso a Scorie Nere Vicano from the Vico Volcanic District (150±4 ka [3, 4]). Previous attempts to chronologically constrain the age of the nearby Monterosi maar, based on ^40^Ar/^39^Ar age determinations on leucite crystals from poorly vesiculated scoria lapilli yielded stratigraphically inconsistent ages, thus evidencing the difficulty of recognize the juvenile fraction in pyroclastic deposits from maar forming eruptions (e.g., [5]). Sample VSM refers to crystals from the ash matrix characterized by plane- to cross-laminated pyroclastic surge deposits exposed (with a thickness of a few meters) in the eastern rim of the VSM maars. The diffuse occurrence of accessory and accidental (clay) ballistic blocks indicates a nearvent location. Interestingly, we recognize the occurrence of cm-sized ballistic blocks from the Tufo Rosso a Scorie Nere Vicano.

**Monte Rocca Romana (MRR)**

The Monte Rocca Romana sample was collected from a few meter-thick lava flow with a ropy top surface, outcropping on the southern slope of the Monte Rocca Romana scoria cone (609 m a.s.l., the highest elevation in the MSVD; Figure 3), located just to the north of Lake Bracciano. This lava has a phonotephritic composition. Previous age determinations on lava samples in the Bracciano area evidenced the occurrence of an intense effusive phase at around ca. 0.3 Ma (e.g., the Cornazzano Lava, dated at 329±4 ka; the Monte Rocca Romana Lava, dated at 317±14; the Vigna di Valle Lava, dated at 284±6 ka [1]).

**Casale Francalancia (CFR)**

The Casale Francalancia eruptive center is located in the eastern Sabatini area, ~3 km to the Sacrofano Caldera eastern rim (Figure 3). The youngest volcanic product cropping out in the area is associated to the 285±1 ka Tufo Giallo di Sacrofano caldera-forming event [1], although no clear stratigraphic relationships with the main eruptive units cropping out in the area exist. The eruptive center consists of a topographic relief derived from associated strombolian and effusive activities. Sample CFR-LF consists of leucite-bearing, phonotephritic lava cropping out in a road cut along the southern slope of the scoria cone.

**Monte Cinghiale (MCG)**

The Monte Cinghiale eruptive center was formed by dominantly effusive activity from the North-Eastern area of the Monti Sabatini District, a relatively peripheral area with respect to the main Sabatini eruptive centers (Figure 3). Sample MCG was collected from a few decimeter-thick lava flow, light grey and highly porphyritic with very abundant leucite phenocrysts up to >1 cm across. No clear stratigraphic relationships with the distal pyroclastic deposits cropping out in the area exist and, as also evidenced by previous works (e.g., [1]), in the MSVD the ages of eccentric strombolian centers span a wide range of time between ~0.3 and 0.09 Ma.

**La Rosta Plinian Fall (LRS)**

We sampled the LRS unit in the eastern Monti Sabatini area (Figure 1), where this pyroclastic fall deposit consists of a ~35 cm thick bed made up of whitish, well vesicular, subaphyric pumice lapilli and includes a basal ~5 cm thick bed with abundant lava lithic inclusions. Glass in the pumice has homogeneous phonolitic composition with SiO_2_ content of ~60-61 wt% and alkali sum of ~13.5-14.5 wt%. This plinian fall rests on top of a brown, mature paleosoil and is sandwiched between a series of decimeter-thick ash layers and black, leucite-bearing (often turned to analcime) scoria lapilli fall deposits, rich in lava lithic inclusions and holocrystalline lithic clasts. Stratigraphically, La Rosta Plinian Fall underlies the Magliano Romano Plinian fall deposit dated at 312±2 ka [1]. In the Eastern Monti Sabatini area, La Rosta Plinian fallout consists of ~35 cm thick bed made up of whitish, well vesicular, subaphyric pumice lapilli and includes a basal ~5 cm thick bed with abundant lava lithic inclusions. The main pumice fallout is sandwiched between a series of decimeter-thick ash layers and black, leucite-bearing (often turned to analcime) scoria lapilli fall deposits, rich in lava lithic inclusions and holocrystalline lithic clasts. By comparing the isopleth maps and published field data for older Plinian Fall deposits from the Southern Sabatini centre [6], we note that the sizes of lithic and pumice clasts of the La Rosta fallout (up to 3-4 cm for pumice and ~1-1.5 cm for lithic clasts) and Fall A and Fall B Plinian fallout deposits [6] display a similar range of variations in the same area. On these grounds, we suggest a possible source area for the La Rosta Plinian fallout broadly corresponding to central Monti Sabatini area.

**VULSINI**

**Monte Bisenzio**

The Monte Bisenzio lava rock-type is subaphyric, with scarce clinopyroxene, leucite and olivine phenocrysts and microphenocrysts; the groundmass is made up of abundant clinopyroxene, leucite, plagioclase and scarce sanidine and magnetite. Chemical composition plots in the shoshonite field of the TAS diagram, close to the trachybasalt field [7], thus being among the least differentiated magmas erupted at Vulsini.

**Lagaccione**

The dune-bedded, phreatomagmatic surge deposits of the Lagaccione tuff ring are exposed around the well preserved maar crater rim on top of co-eruptive Hawaiian-Strombolian spatter and scoria fall deposits. The latter are trachybasaltic in composition, again representing a relatively poorly differentiated magma.

REFERENCES

[1] Sottili, G. *et al.* Geochronology of the most recent activity in the Sabatini Volcanic District, Roman Province, central Italy. ***J. Volcanol. Geotherm. Res.***  **196**, 20-30 (2010).

[2] Nappi, G. & Mattioli, M. Evolution of the Sabatinian Volcanic District (central Italy) as inferred by stratigraphic successions of its northern sector and geochronological data. *Per. Mineral.* **72**, 79–102 (2003).

[3] De Rita, D., Funiciello, R., Corda, L., Sposato, A. & Rossi, U. Volcanic Units. In: Di Filippo, M., (Ed.), Sabatini Volcanic Complex, *Quad. Ric. Sci.* 114, Progetto Finalizzato Geodinamica C.N.R., Roma, 33-79 (1993).

[4] Laurenzi, M.A., & Villa, I.M. 40Ar/39Ar chronostratigraphy of the Vico ignimbrites. *Per. Mineral.* **56**, 285–293 (1987).

[5] Valentine, G.A., Sottili, G., Palladino, D.M. & Taddeucci J. Tephra ring interpretation in light of evolving maar-diatreme concepts: Stracciacappa maar (central Italy), *J. Volcanol. Geotherm. Res.* **308**, 19-29 (2015). DOI:[10.1016/j.jvolgeores.2015.10.010](http://dx.doi.org/10.1016/j.jvolgeores.2015.10.010)

[6] Sottili, G., Palladino, D.M. & Zanon, V. Plinian activity during the early eruptive history of the Sabatini Volcanic District, Central Italy. *J. Volcanol. Geotherm. Res.* 135, 361-379 (2004).

[7] Palladino, D.M., Agosta, E., Freda, C., Spaziani, S. & Trigila, R. Geo-petrographic and volcanological study of Southern Vulsini: The Valentano–Marta–La Rocca sector. *Memorie Descrittive della Carta Geologica d’Italia* **49**, 255–276 (1994).
